# Multi-omics classification of acute myeloid leukemia guides drug combinations to overcome Venetoclax resistance

**DOI:** 10.20517/cdr.2025.228

**Published:** 2026-03-26

**Authors:** Runyu Yang, Hui Feng, Mengyao Zhang, Yi Liu, Minna Luo, Ruimin Liu, Kaiyao Wang, Qijing Li, Wenjuan Wang, Jing Chen, Yue Du, Jiayi Xiao, Bingyu Yang, Fan Niu, Pengcheng He

**Affiliations:** ^1^Department of Hematology, The First Affiliated Hospital of Xi’an Jiaotong University, Xi’an 710061, Shaanxi, China.; ^2^Shaanxi University of Chinese Medicine, Xi’an-Xianyang New Economic Zone, Xi’an 712046, Shaanxi, China.; ^#^These authors contributed equally to this work and share first authorship.

**Keywords:** Acute myeloid leukemia, multi-omics, molecular subtypes, Venetoclax resistance, precision medicine

## Abstract

**Aim:** Acute myeloid leukemia (AML) is an aggressive hematological malignancy. Conventional risk stratification in AML fails to predict patient responses to targeted therapies such as Venetoclax, hindering precision medicine and the development of strategies to overcome drug resistance.

**Methods:** We established an integrated multi-omics framework incorporating messenger RNA (mRNA)/long non-coding RNA (lncRNA) expression, DNA methylation, copy number alterations, and somatic mutation data. Using nine complementary clustering algorithms, we identified molecular subtypes in a discovery cohort and validated them in independent external cohorts. The multi-omics classification subsequently guided the screening of high-risk subtypes specific sensitizers to Venetoclax, with candidate efficacy validated through *in vitro* and *in vivo* experiments.

**Results:** We identified three molecularly distinct AML subtypes with unique clinical features, a classification that was subsequently validated in independent external cohorts. Cluster 2 demonstrated the most favorable prognosis, while Cluster 3, characterized by high tumor protein 53 (TP53) mutation frequency and significant immune infiltration, exhibited the poorest outcomes and pronounced resistance to Venetoclax. Multi-omics-guided drug screening revealed that Cluster 3 displays particular sensitivity to both Elesclomol and the clinically available proteasome inhibitor Bortezomib. Through comprehensive *in vitro* and *in vivo* validation, we demonstrated that both agents significantly enhance the therapeutic efficacy of Venetoclax. Of particular translational relevance, the Venetoclax-Bortezomib combination leverages an agent with an established safety profile, offering a readily implementable strategy for near-future clinical translation.

**Conclusion:** Our study establishes a novel multi-omics classification system that provides a robust foundation for investigating biological heterogeneity, elucidating resistance mechanisms, and developing effective combination strategies to achieve personalized therapy in AML.

## INTRODUCTION

Acute myeloid leukemia (AML) represents a highly aggressive hematologic malignancy characterized by remarkable heterogeneity and diverse molecular features, which collectively contribute to substantial variations in patient prognosis and therapeutic responses^[1]^. This heterogeneity poses a fundamental challenge to the implementation of precision medicine. Current clinical approaches to risk stratification, primarily based on genomics and cytogenetics, have only partially addressed this complexity^[2]^. The inadequacy of these conventional systems manifests in several critical and interconnected limitations. First, they often provide an imprecise prognosis, diverging from actual clinical outcomes and leading to suboptimal treatment choices^[3]^. More fundamentally, they fail to adequately capture the underlying biological characteristics^[4]^ and distinct pathogenic processes of AML, as they lack integration across molecular layers^[5-7]^. This critical gap in understanding the disease’s functional biology directly undermines their ability to reliably predict responses to targeted therapies such as Venetoclax; consequently, most identified molecular alterations still lack effective and predictable targeted interventions. This gap between molecular cataloguing and therapeutic insight limits the utility of current stratification in AML precision medicine. Consequently, there is an urgent need to establish more sophisticated stratification frameworks that can decipher the biological distinctions among AML subtypes and provide a theoretical foundation for enhancing targeted therapy.

Advances in high-throughput technologies have ushered in a new era of understanding AML heterogeneity at multiple levels^[8]^. However, reliance on single-omics approaches is insufficient as they offer only a fragmented view^[9-11]^. Therefore, the integration of multi-omics data is essential to construct a holistic biological model of the disease. Concurrently, ongoing advances in artificial intelligence (AI) have driven the development of numerous machine learning algorithms^[12,13]^. These computational tools now make it feasible to integrate complex, high-dimensional datasets, allowing us to accurately identify molecularly coherent and clinically relevant disease subtypes.

In this study, we employed an AI-driven approach to integrate five dimensions of multi-omics data, including messenger RNA (mRNA) expression, long non-coding RNA (lncRNA) expression, DNA methylation, copy number alterations, and somatic mutations. By utilizing nine distinct machine learning algorithms, we identified three AML subtypes with distinct prognostic outcomes. The robustness of this classification was validated across multiple independent cohorts. Notably, Cluster 3 was newly identified as a poor-prognosis subtype exhibiting marked resistance to Venetoclax, characterized by high tumor protein 53 (TP53) mutation frequency, hyperactive mitochondrial metabolism, monocytic differentiation patterns, and activation of chronic inflammatory signaling. Moving beyond classification, we conducted multi-omics-subtypes guided drug screening and discovered that Cluster 3 patients demonstrate heightened sensitivity to both Elesclomol and the clinically available proteasome inhibitor, Bortezomib^[14]^. Through comprehensive *in vitro* and *in vivo* validation, we confirmed that both agents significantly enhance the therapeutic efficacy of Venetoclax without increasing toxicity to normal cells from healthy donors. Of these, the Venetoclax-Bortezomib combination is of particular translational interest. The well-established safety profile and extensive clinical use of Bortezomib in hematological malignancies could enable a more rapid path to clinical application for these high-risk AML subtypes. Ultimately, we established a novel multi-omics-based classification system for AML patient stratification and developed an interactive web platform to facilitate clinical implementation. Our work thus delivers a comprehensive diagnostic and therapeutic strategy, providing a robust foundation for investigating biological heterogeneity, elucidating resistance mechanisms, and developing effective combination strategies to achieve personalized therapy in AML.

## METHODS

### Collection and preprocessing of multi-omics datasets

We initiated data collection from The Cancer Genome Atlas-Acute Myeloid Leukemia (TCGA-LAML) cohort using the R package easyTCGA (version 0.0.1.6000) to procure mRNA and lncRNA expression profiles. Transcriptome data were transformed to log2 [transcripts per million (TPM) + 1] to enhance comparability. Simultaneously, clinical-pathological information, copy number segments, and DNA methylation profiles were retrieved from the UCSC Xena database (University of California Santa Cruz cancer database, https://xenabrowser.net/). Somatic mutation profiles were sourced from the cBioPortal database (https://www.cbioportal.org/). Normalized transcriptome data and clinical metadata from the Beat AML cohort were accessed from the BeatAML2 database (https://biodev.github.io/BeatAML2/). Utilizing the R package GEOquery (version 2.66.0), we retrieved datasets GSE10358^[15]^, GSE71014^[16]^, GSE14468^[17]^, GSE37642-GPL570, and GSE37642-GPL96^[18]^. Data normalization was performed using the “normalizeBetweenArrays” function from the R package limma (version 3.54.2). For ID conversion, hgu133plus2.db (version 3.13.0) was used for the GSE10358, GSE14468, and GSE37642-GPL570 datasets. illuminaHumanv4.db (version 3.13.0) was applied to GSE71014, while hgu133a.db (version 3.13.0) was used for GSE37642-GPL96. In cases where multiple probe IDs mapped to a single gene symbol, median values were computed. Raw transcript counts data for mono-AML and prim-AML bulk transcriptomes were sourced from GSE132511^[19]^. Detailed information on the different datasets can be found in Supplementary Table 1.

### Integration and subtype construction of TCGA-LAML cohort raw data

#### Integration of raw data

Initially, we integrated multi-omics data from TCGA-LAML patients, including mRNA expression levels, lncRNA expression levels, DNA methylation profiles, copy number alterations, and somatic mutation data. Given partial data availability across these multi-omics layers, we consolidated data from 121 patients.

#### Subtype construction

To construct molecular subtypes that closely align with clinical features with precision and speed, we utilized the “getElites” function from the R package MOVICS (version 0.99.17)^[20]^. This function employs Cox regression analysis to select and extract factors most significantly associated with overall survival (OS), considering factors with a *P*-value less than 0.001. Ultimately, 44 mRNA [Supplementary Table 2], 90 lncRNA [Supplementary Table 3], 313 copy number alteration (CNA) [Supplementary Table 4], and 13 DNA methylation sites [Supplementary Table 5] were included. Additionally, 18 genes with mutation frequencies exceeding 5% were incorporated into the multi-omics analysis. While survival-associated features were used to ensure clinical relevance, rigorous external validation was subsequently performed to ensure the generalizability of the subtypes and mitigate potential overfitting bias (see Section “External validation of subtype classification and protein-protein interaction network construction”). The “getMOIC” function was used to subtype patients using nine algorithms: “Similarity Network Fusion (SNF)”, “Perturbation Clustering for Data Integration (PINSPlus)”, “NEighborhood-based Multi-Omics clustering (NEMO)”, “Cluster-of-Clusters Analysis (COCA)”, “Low-Rank Approximation-based clustering (LRAcluster)”, “(Consensus Clustering (ConsensusClustering)”, “Integrative Non-negative Matrix Factorization (IntNMF)”, “Cancer Integration via Multikernel Learning (CIMLR)”, and “Multi-Omics Clustering (MoCluster)”. The “getConsensusMOIC” function was applied to integrate results from multiple subtype algorithms and evaluate subtype quality using the “getSilhouette” function.

#### Subtype visualization and clinical data analysis

The “getMoHeatmap” function was employed to visualize classification results, and the “compSurv” function was used to assess survival differences among different subtype patients. The “compMut” function was used to compare gene mutation frequency differences across different clusters and visualize the results.

### External validation of subtype classification and protein-protein interaction network construction

To critically evaluate the robustness of our multi-omics classification and address the risk of circular reasoning inherent in survival-based feature selection, we applied the predefined clustering template to several independent cohorts. These cohorts served as External cross-platform verification where the molecular features and clinical outcomes were entirely independent of the discovery phase, providing an unbiased assessment of the classification’s prognostic and biological stability. Initially, we utilized the “runDEA” function with the limma algorithm to conduct differential gene expression analysis (DEA) for each cluster. The top 100 upregulated genes from each cluster were extracted as template files [Supplementary Table 6]. Subsequently, the “run nearest template prediction (runNTP)” function was employed for external dataset prediction. To analyze and visualize prognostic differences among patients within each cluster across different external datasets, we utilized the R packages survival (version 3.5-5) and survminer (version 0.4.9). Simultaneously, we input the top 100 upregulated genes from each cluster into the Search Tool for the Retrieval of Interacting Genes (STRING) database (https://cn.string-db.org/)^[21]^ to construct protein-protein interaction (PPI) networks, setting the interaction score threshold to 0.7. We utilized the R package igraph (version 1.5.0) to construct network modules and performed enrichment analysis of genes within each module using the R package clusterProfiler (version 4.10.0)^[22]^.

### Analysis of biological features across different clusters at the single-cell level

#### Clustering and visualization

We downloaded the GSE116256 dataset from the Gene Expression Omnibus (GEO) database^[23]^, comprising bone marrow samples from 12 newly diagnosed AML patients and 4 healthy donors. Single-cell level analysis was conducted using the R package Seurat (version 4.3.01). The “CreateSeuratObject” function was employed to generate the SeuratObject. Data normalization was performed using LogNormalize with a scale factor of 10,000. Highly variable genes were identified using the “FindVariableFeatures” function with the parameter “nfeatures” set to 2,000. Principal component analysis (PCA) was conducted on the dataset using the “RunPCA” function. The R package harmony (version 1.2.0) was used to mitigate batch effects between samples. We utilized the top 30 principal components to construct a k-nearest neighbor graph using the “FindNeighbors” function. Subsequently, for visualization purposes, data dimensions were reduced using the uniform manifold approximation and projection (UMAP) algorithm via the “RunUMAP” function. Cell annotation across different cell types was performed according to the classification system described by van Galen *et al*.^[23]^.

#### Single-cell and bulk-transcriptome integration analysis

The function “runNTP” from the R package MOVICS was utilized to assign each AML cell from GSE116256 to different clusters. We selected the top 100 upregulated marker genes from each cluster mentioned in section “Integration and subtype construction of TCGA-LAML cohort raw data” as pathways. The activity of these cluster-specific signatures was then quantified using the AUCell algorithm (version 1.22.0). This rank-based approach calculates the area under the curve (AUC) for the recovery of marker genes within the top expression rankings of each individual cell, ensuring a stable assessment of cluster identities across the single cell landscape. Additionally, marker genes for different types of AML cell types [hematopoietic stem cell (HSC)-like, progenitor-like, granulocyte-macrophage progenitor (GMP)-like, promonocyte-like, monocyte-like, and conventional dendritic cell (cDC)-like] were obtained from van Galen *et al*.^[23]^. Using these markers, the single-sample gene set enrichment analysis (ssGSEA) algorithm was applied to TCGA-LAML samples. The resulting scores for each AML cell type were then compared across different clusters.

#### Analysis of differentiation trajectories of AML cells

The R package Monocle2 (version 2.26.0)^[24]^ was employed to analyze the differentiation trajectory of cells within Cluster 3, identifying cell states based on pseudotime. Lastly, we identified genes most significantly associated with pseudotime changes in the differentiation of AML cells in Cluster 3. Using the R package clusterProfiler (version 4.10.0), we performed enrichment analysis on genes within each module.

### Assessment of immune infiltration levels in different clusters

The CIBERSORT algorithm was utilized to evaluate the abundance of immune cell infiltration across different clusters, with the results visualized using heatmaps. The R package TCellSI (https://guolab.wchscu.cn/TCellSI/)^[25]^ was employed to assess T cell states (quiescent, regulatory, proliferative, helper, cytotoxic, progenitor exhaustion, terminal exhaustion, and senescence) in patient samples. The R package tigeR^[26]^, a robust tool for identifying biomarkers predictive of immunotherapy efficacy and constructing predictive models, was used to predict patient responses to immunotherapy across multiple cohorts.

### Drug sensitivity analysis across different clusters

Drug response data (AUC values) and the list of Inhibitor Families for *ex vivo* patient samples in the Beat AML cohort were obtained from the BeatAML2 database (https://biodev.github.io/BeatAML2/). After normalizing the median AUC values for various inhibitors across different clusters, the R package pheatmap (version 1.0.12) was utilized for visualization. Transcriptomic profiles of different cell lines from the Cancer Cell Line Encyclopedia (CCLE) database can be accessed via (20Q4: https://depmap.org/portal/). The function “runNTP” from the R package MOVICS was employed to assign each AML cell line to its respective cluster.

### Cell culture

The MOLM13 cell line was obtained from DSMZ (German Collection of Microorganisms and Cell Cultures); M07e and U937 from ATCC (American Type Culture Collection); HEL and Kasumi-1 from Procell Life Science & Technology Co., Ltd.; and OCI-AML3 from Nanjing Cobioer Biosciences Co., Ltd., China. MOLM13, M07e, U937, HEL, and OCI-AML3 cell lines were cultured in suspension using RPMI-1640 medium (HyClone) supplemented with 10% fetal bovine serum (FBS; Newzerum). Kasumi-1 cells were cultured in RPMI-1640 medium containing 20% FBS, 1% sodium pyruvate (100 mM, Solarbio), and 1% L-glutamine (200 mM, Solarbio). All cell lines were maintained at 37 °C with CO_2_.

### Construction of Venetoclax-resistant cell line and RNA sequencing

To generate Venetoclax-resistant cell lines, MOLM13 cells were cultured in medium containing increasing concentrations of Venetoclax (5-1,000 nmol/L, Selleck) over a period of more than 3 months to achieve complete drug resistance (MOLM13/Vene). RNA sequencing (RNA-seq) analysis was performed on two cell lines, MOLM13 and MOLM13/Vene, with three replicates for each. Cells were lysed with TRIzol and stored at -80 °C. RNA-seq was conducted by Beijing Tsingke Biotech Co., Ltd. Sequence alignment was performed using Hisat2 with the reference genome GRCh38 (release 112). Gene expression was quantified using StringTie, and expression levels were normalized using fragments per kilobase of transcript per million fragments mapped (FPKM) and TPM for subsequent analysis.

### Isolation of primary AML cells from patient bone marrow

All procedures involving human samples were conducted after approval by the Ethics Committee of the First Affiliated Hospital of Xi’an Jiaotong University (Approval No. LLSBPJ-2024-493), and written informed consent was obtained from all patients. Primary cells were isolated from the bone marrow of patients with newly diagnosed or relapsed/refractory AML. Detailed clinical information for AML patients can be found in Supplementary Table 7. Bone marrow samples were mixed with an equal volume of phosphate-buffered saline (PBS) and 6% hydroxyethyl starch (TBDscience) and incubated for 25 min to allow red blood cell sedimentation. The supernatant was collected and centrifuged at 1,500 rpm for 5 min. The supernatant was discarded, and the cell pellet was resuspended in 1 mL of PBS. To lyse the red blood cells, 3 mL of Red Blood Cell Lysis Buffer (Solarbio) was added, followed by a 5-minute incubation and subsequent centrifugation at 1,500 rpm for 5 min. The resulting cell pellet was resuspended in RPMI-1640 medium containing 10% FBS and cultured at 37 °C with 5% CO_2_. The isolated cells were then used for subsequent experiments.

### Drug treatment and IC_50_ measurement

Cell viability was assessed using the Cell Counting Kit-8 (CCK-8) (Dojindo® Laboratories). Cells were seeded into 96-well plates at a density of 3 × 10^5^ cells/mL and exposed to gradually increasing concentrations of Venetoclax, Elesclomol (in the presence of 100 nM CuCl_2_, MedChemExpress), or Bortezomib (MedChemExpress). Each concentration for each cell line was tested in at least three technical replicates. After co-incubation for 24-48 h, 10 µL of CCK-8 solution was added to each well and incubated for an additional 3 h. Absorbance was measured at 450 and 690 nm using a microplate reader. Absolute viability values were normalized to the control treatment, and the resulting data were expressed as a percentage of the control viability. The half-maximal inhibitory concentration (IC_50_) values were determined using nonlinear regression analysis of log [inhibitor] *vs.* response (three parameters) in GraphPad Prism v9.0.

### Multi-drug combination analysis

OCI-AML3, U937, and MOLM13/Vene cells were seeded into 96-well plates at a density of 3 × 10^5^ cells/mL and exposed to increasing concentrations of Venetoclax and varying concentrations of Elesclomol or Bortezomib. Each concentration for each cell line was tested in at least three technical replicates. After a 24-hour co-incubation period, cell viability was assessed using the CCK-8 assay to determine relative cell viability at different concentrations. For the analysis of drug combination effects, SynergyFinder 3.0 (https://synergyfinder.fimm.fi/) was employed^[27]^. The four-parameter logistic regression (LL4) was used as the curve-fitting algorithm, and the consensus non-negative matrix factorization (cNMF) algorithm was applied for the estimation of outlier measurements. SynergyFinder calculated the expected drug combination synergy scores based on the zero interaction potency (ZIP) reference model. A synergy score greater than 10 indicates a likely synergistic interaction between the two drugs.

### FACS analysis of apoptosis

OCI-AML3, U937, MOLM13/Vene, and patient-derived primary AML cells were seeded into 24-well plates at a density of 5 × 10^5^ cells/mL. The cells were then treated with increasing concentrations of Venetoclax, Elesclomol or Bortezomib (MedChemExpress). After 24 h, the cells were harvested and centrifuged at 1,500 rpm for 5 min. The supernatant was discarded, and the cell pellet was resuspended in 100 μL of fetal calf serum (FCS) buffer. Apoptosis was detected by staining the cells with 5 μL of Annexin V and 10 μL of propidium iodide (PI, Biolegend®), followed by incubation in the dark at room temperature (20-25 °C) for 15 min. Subsequently, 400 μL of binding solution was added to each sample. Flow cytometry was conducted immediately using the BD FACS Canto II® flow cytometer, and the data were analyzed and visualized with FlowJo software (version 10). The survival cell ratios in each treatment group were normalized to the control group.

### *In vivo* evaluation of combined therapeutic efficacy in a xenograft AML mouse model

Female NOD scid gamma (NSG) mice aged 6-8 weeks were purchased from Shanghai Model Organisms Center, Inc. A xenograft mouse model was established by intravenous injection of 2 × 10^4^ MOLM13/Vene cells via the tail vein into NSG mice. All animal experimental procedures were conducted in accordance with institutional guidelines and approved by the Laboratory Animal Center of Xi’an Jiaotong University (Approval No. XJTUAE2024-2165).

For the *in vivo* combination therapy of Venetoclax and Elesclomol, recipient mice were administered Venetoclax (50 mg/kg) via intraperitoneal injection every two days from day 5 to day 21. Venetoclax was formulated in 15% Cremophor EL (MedChemExpress). Elesclomol (10 mg/kg) was reconstituted in saline and administered every three days from day 5 to day 21. On day 23, all mice were euthanized, and human leukemia cells were harvested from bone marrow and spleen. For bone marrow isolation, tibiae and femora were dissected and flushed with RPMI-1640. Spleens were minced and homogenized, followed by red blood cell lysis using Red Blood Cell Lysis Buffer. After cell counting, bone marrow specimens and splenocytes were stained with anti-human CD45 (Biolegend) to label leukemia cells, and the proportion of leukemic cells was quantified by flow cytometry.

For the *in vivo* combination therapy of Venetoclax and Bortezomib, recipient mice received Venetoclax (50 mg/kg) intraperitoneally every two days from day 5 to day 21. Bortezomib (1 mg/kg) was dissolved in dimethyl sulfoxide (DMSO) and diluted in saline (final DMSO concentration 0.5%), and administered every three days from day 5 to day 21. Tumor burden was assessed as described above.

### Statistical analysis

No statistical methods were used to predetermine the sample size. For statistical comparisons, log-rank tests were used to calculate *P*-values for Kaplan-Meier survival curves. Differences between two groups for continuous variables were analyzed using unpaired Student’s *t*-test, while differences among three or more groups were assessed with the Kruskal-Wallis test. For categorical variables, chi-square tests and Fisher’s exact tests were conducted. To compare gene mutation frequencies between clusters, permutation tests were performed. Statistical significance was defined as *P* < 0.05, with ^*^P < 0.05, ^**^P < 0.01, ^***^P < 0.001, and ns = not significant. All bioinformatics analyses were performed using R software (version 4.2.2). Visualizations were created using R software and GraphPad Prism 9.

## RESULTS

### Identification of three distinct clusters and evaluation of their clinical indicators

**Figure 1:**
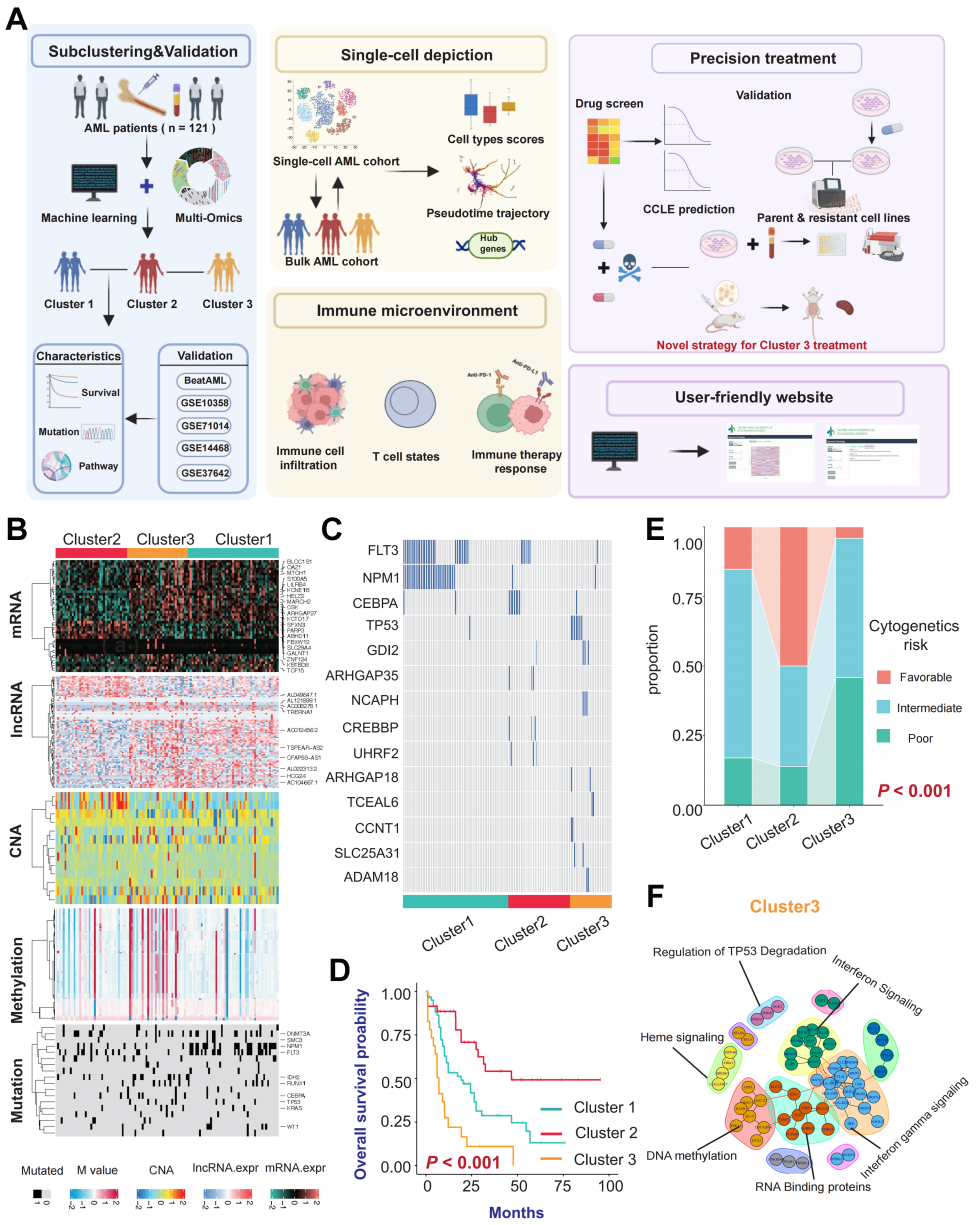
Identification of three distinct clusters and evaluation of different clinical indicators. (A) Workflow of this study; (B) Heatmap of multi-omics characteristics for the three subtypes; (C) Differences in gene mutation frequencies among the three clusters; (D) Kaplan-Meier survival curves illustrate differences in OS among patients with different clusters (*P* < 0.001); (E) Comparison of the existing AML cytogenetic risk standards with the three subtypes identified through clustering in this study; (F) PPI network interactions of upregulated differential genes in Cluster 3, showing enriched pathways for different gene modules. Log-rank tests were used to calculate *P*-values for Kaplan-Meier survival curves, and categorical variables were analyzed using the chi-square test or Fisher’s exact test. The color scheme is maintained throughout the study where turquoise represents Cluster 1, red represents Cluster 2, and yellow represents Cluster 3. Created in BioRender. Yang, R. (2026) https://BioRender.com/w0nln0a. OS: Overall survival; AML: acute myeloid leukemia; PPI: protein-protein interaction; PD-1: programmed cell death protein 1; PD-L1: programmed cell death ligand 1; CCLE: Cancer Cell Line Encyclopedia; mRNA: messenger RNA; lncRNA: long non-coding RNA; CNA: copy number alteration; TP53: tumor protein 53.

We integrated multi-omics data from 121 patients in the TCGA-LAML cohort. Using nine different clustering algorithms, we categorized the patients into three predefined multi-omics subtypes [Figure 1A]. These were subsequently consolidated into robust classifications through integrated consensus [Supplementary Figure 1A-C]. The distribution of the multi-omics data across the three clusters is shown in Figure 1B. We observed that Cluster 3 exhibited higher levels of DNA methylation, with a higher proportion of patients harboring TP53 mutations also classified into Cluster 3. In Cluster 2, a higher proportion of patients had CCAAT/enhancer-binding protein alpha (CEBPA) mutations, while Cluster 1 had a higher prevalence of FMS-like tyrosine kinase 3 (FLT3) and nucleophosmin 1 (NPM1) mutations [Figure 1B and C, Supplementary Figure 1D]. Notably, the classification was supported by high concordance among the data layers, as subtype boundaries were consistently reinforced by parallel signals in the genome, epigenome, and transcriptome. This multi-layered stability ensures that the identified molecular states are robust and less susceptible to noise inherent in any single platform. Next, we compared the prognosis of patients across these three clusters, revealing that patients in Cluster 2 had better OS, whereas those in Cluster 3 had the worst prognosis [Figure 1D]. This indicates that patients in different multi-omics clusters face distinct clinical outcomes. A Sankey diagram compared the current AML cytogenetic risk standards with the three clusters identified in our study. We found that the favorable risk group was most prevalent in Cluster 2, while the poor risk group was most prevalent in Cluster 3, corroborating our survival analysis results [Figure 1E]. To gain a deeper understanding of the biological behavior differences among the three subtypes, we calculated the differentially expressed genes (DEGs) for each cluster compared to the other two. Subsequently, we selected the top 100 upregulated DEGs to construct a PPI network. We performed Kyoto Encyclopedia of Genes and Genomes (KEGG) enrichment analysis on different modules within the PPI network to identify cluster-specific activated signaling pathways. The results showed that the upregulated gene modules in Cluster 3 were enriched primarily in Interferon Signaling, TP53, and DNA methylation pathways [Figure 1F]. Meanwhile, the upregulated gene modules in Cluster 1 were enriched in DNA Replication and G protein-coupled receptor (GPCR) ligand binding [Supplementary Figure 1E], and those in Cluster 2 were enriched in G-protein activation and Golgi-to-endoplasmic reticulum (ER) retrograde transport [Supplementary Figure 1F].

### Reproduction of AML multi-omics clusters results in external cohorts

**Figure 2:**
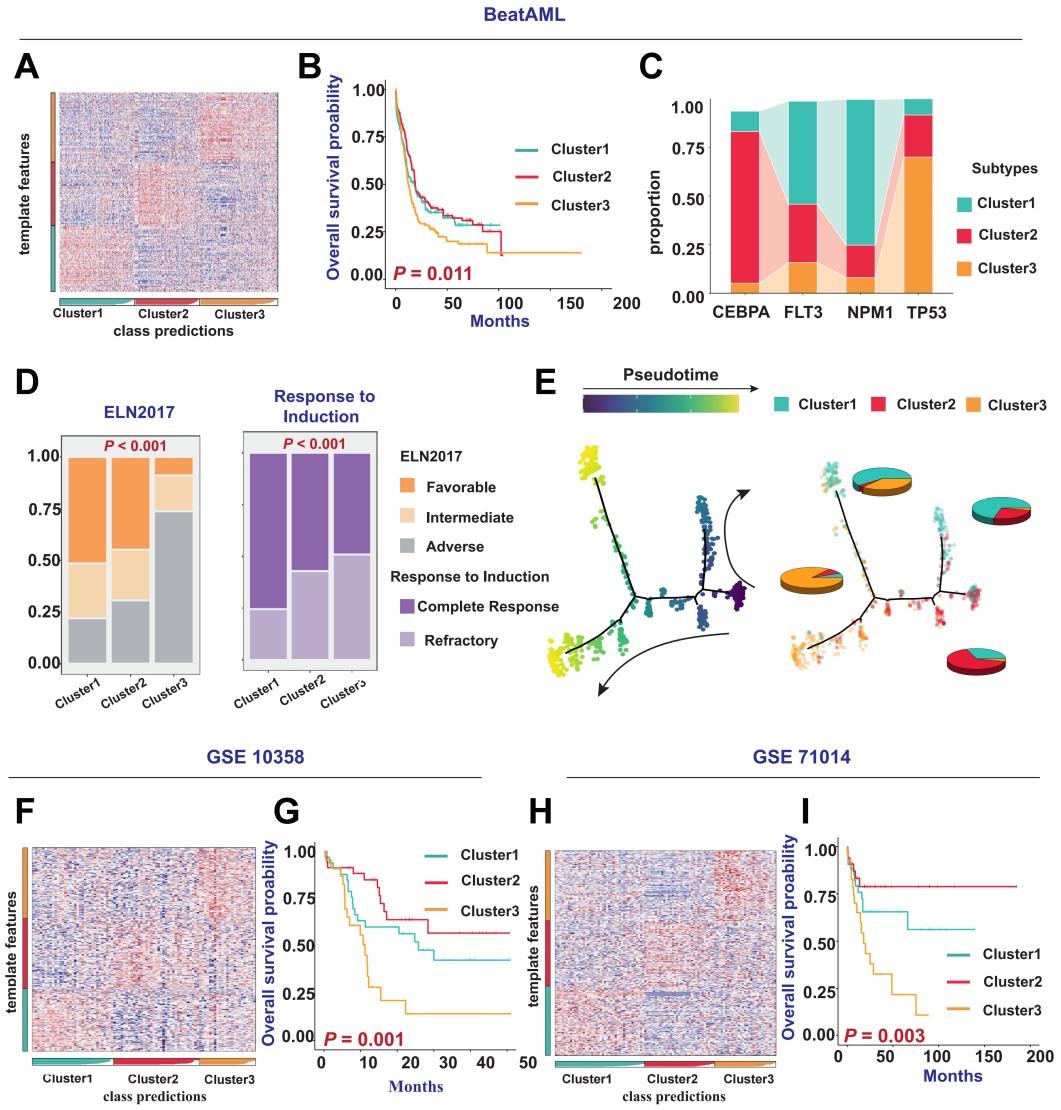
Reproduction of AML molecular subtyping results in external cohorts. (A) Prediction and classification of patient samples using NTP in the BEAT AML cohort; (B) Kaplan-Meier survival curves illustrating differences in OS among patients with different clusters in the BEAT AML cohort (*P* = 0.011); (C) Comparison of *CEBPA*, *FLT3*, *NPM1*, and *TP53* gene mutations with the three clusters; (D) Bar chart describing the relationship between clinical indicators such as ELN 2017 risk categories, patient responses to induction therapy and the three clusters in the BEAT AML cohort; (E) Simulation of patient differentiation trajectories in different clusters within the BEAT AML cohort; (F and H) Prediction and classification of patient samples using NTP in the GSE10358 and GSE71014 cohorts; (G and I) Kaplan-Meier survival curves showing differences in OS among patients with different subtypes in the GSE10358 cohort (*P* = 0.001) and GSE71014 cohort (*P* = 0.003). Log-rank tests were used to calculate *P*-values for Kaplan-Meier survival curves, and categorical variables were analyzed using the chi-square test or Fisher’s exact test. AML: Acute myeloid leukemia; NTP: nearest template prediction; OS: overall survival; CEBPA: CCAAT/enhancer-binding protein alpha; FLT3: FMS-like tyrosine kinase 3; NPM1: nucleophosmin 1; TP53: tumor protein 53; ELN: European LeukemiaNet.

To further validate the generalizability and robustness of the aforementioned multi-omic clusters, we reproduced the subtyping results in several external cohorts. We applied the multi-omics classification strategy to predict the BEAT AML cohort [Figure 2A] and found that patients in Cluster 3 had the poorest prognosis [Figure 2B]. A Sankey diagram showed that patients with FLT3 and NPM1 mutations were more frequently classified into Cluster 1, those with CEBPA mutations were predominantly found in Cluster 2, and patients with TP53 mutations were most commonly assigned to Cluster 3 [Figure 2C]. These findings were highly consistent with those observed in the TCGA-LAML cohort and were further corroborated by the GSE14468 cohort [Supplementary Figure 2A and B]. Additionally, we found a higher proportion of patients with high-risk cytogenetic subtypes and poor treatment outcomes in Cluster 3 [Figure 2D], consistent with findings from the TCGA-LAML and GSE14468 cohorts [Supplementary Figure 2C]. To determine the evolutionary relationships among patients in different clusters, we applied the monocle trajectory analysis concept from single-cell transcriptomics, treating each patient as a single cell and projecting the cluster information onto the subtype evolution trajectory. The results showed that the horizontal axis of the trajectory represented overall risk evolution. Cluster 2 (best prognosis) and Cluster 3 (worst prognosis) were located at opposite ends of the trajectory, whereas Cluster 1 occupied an intermediate position. Each cluster maintained good intra-cluster consistency and inter-cluster differentiation [Figure 2E]. In addition to the Beat AML cohort, we also performed predictions and clustering on patient samples in four other cohorts, including GSE10358, GSE71014, and GSE37642 (GPL-96 and GPL-570). Prognostic survival analyses consistently showed that the prognosis for patients in Cluster 3 was the poorest (all *P* < 0.05) [Figure 2F-I, Supplementary Figure 2D-G].

### Single-cell analysis of biological features across different clusters

**Figure 3:**
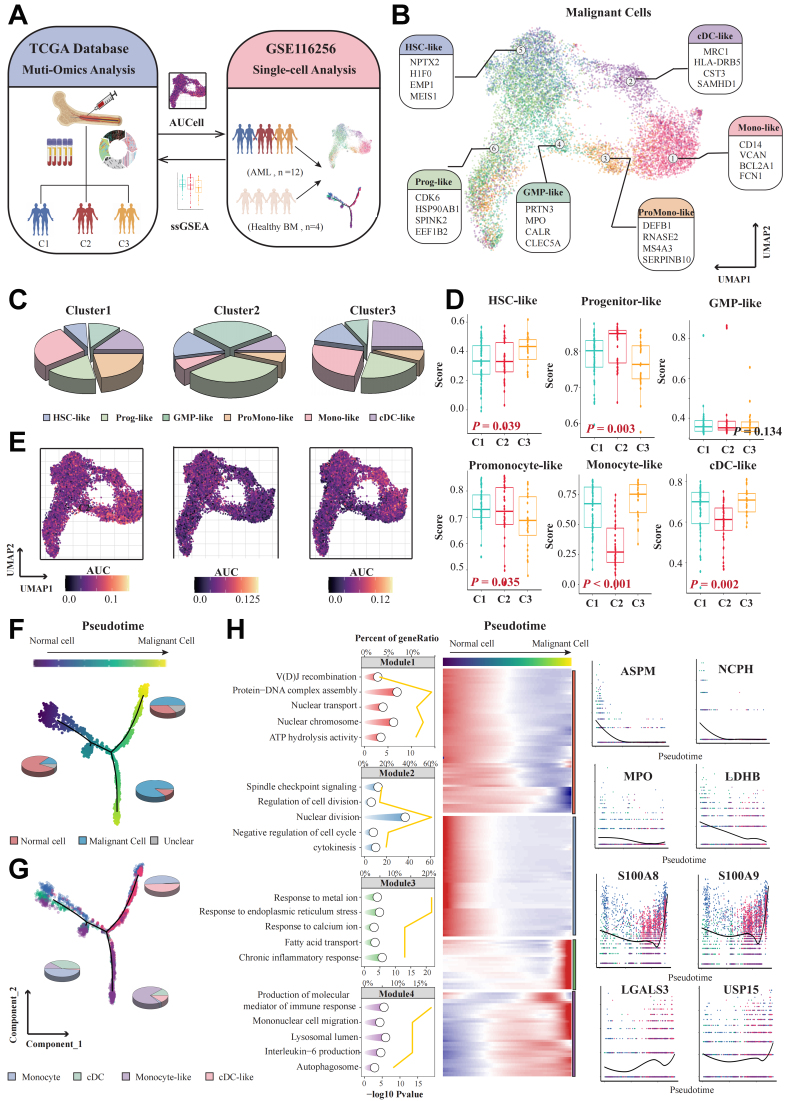
Single-cell depiction of three AML subtypes. (A) Workflow of integrated analysis of bulk transcriptomics and single-cell transcriptomics. The UMAP plot illustrates that 12 newly diagnosed AML samples from GSE116256 have been comprehensively annotated into 6 distinct cell types; (B) Each color on the plot corresponds to a specific cell type, as indicated in the legend. Cell annotations are based on previous research by van Galen *et al*.^[23]^; (C) Pie chart showing the proportion of cell types in the three different subtypes; (D) Boxplot of different AML cell types scores: HSC-like (*P* = 0.039), progenitor-like (*P* = 0.003), GMP-like (*P* = 0.134), promonocyte-like (*P* = 0.035), monocyte-like (*P* < 0.001), cDC-like (*P* = 0.002) in patients of different clusters from the TCGA-LAML cohort; (E) AUCell scores representing the activation levels of pathways comprising upregulated genes in the three clusters, visualized with a gradient color scheme; (F) AUCell scores representing the activation levels of pathways comprising upregulated genes in the three clusters, visualized with a gradient color scheme. Pseudotime trajectory plot showing the transformation of cells from normal to malignant in the Cluster 3 subtype, visualized by pseudotime; (G) Visualization of cells by cell type; (H) Enrichment analysis of representative genes from different modules along the pseudotime, visualized by a star plot. Heatmap showing significant changes in four modules along pseudotime. Expression of representative genes (*ASPM*, *NCPH*, *MPO*, *LDHB*, *S100A8*, *S100A9*, *LGALS3*, *USP15*) along the pseudotime trajectory. Differences among three or more groups were assessed with the Kruskal-Wallis test. Created in BioRender. Yang, R. (2026) https://BioRender.com/2cn7hfa. AML: Acute myeloid leukemia; UMAP: uniform manifold approximation and projection; HSC: hematopoietic stem cell; GMP: granulocyte-macrophage progenitor; cDC: conventional dendritic cell; TCGA-LAML: The Cancer Genome Atlas-Acute Myeloid Leukemia; ASPM: abnormal spindle-like microcephaly-associated protein; NCPH: non-SMC condensin I complex subunit H; MPO: myeloperoxidase; LDHB: lactate dehydrogenase B; LGALS3: lectin galactoside-binding soluble 3; USP15: ubiquitin specific peptidase 15; ssGSEA: single-sample gene set enrichment analysis; BM: bone marrow; AUC: area under the curve.

We then performed an integrative analysis between single-cell and bulk transcriptome data [Figure 3A]. Initially, we categorized each cell using 300 cluster-specific markers and allocated each patient to different clusters based on the proportion of cells from each cluster within their sample [Supplementary Figure 3A]. We observed a higher proportion of FLT3 and NPM1 mutations in patients assigned to Cluster 1. In contrast, a higher proportion of TP53 mutations was observed in patients assigned to Cluster 3 [Supplementary Figure 3B], which is highly consistent with the phenomena observed in the bulk transcriptome cohort. Next, using marker genes for different types of AML cells provided by van Galen *et al*.^[23]^, we annotated malignant AML cells into six different cell types [Figure 3B]. The pie charts illustrate that progenitor-like and GMP-like cells are predominant in Cluster 2, while monocyte-like and cDC-like cells are predominant in Cluster 3 [Figure 3C]. Subsequently, we applied the ssGSEA algorithm to evaluate the scores of the six AML cell types in different patients from the TCGA-LAML cohort. We found that, similar to the single-cell transcriptome cohort, patients classified into Cluster 3 in the TCGA-LAML cohort had higher scores of monocyte-like (*P* < 0.001) and cDC-like (*P* = 0.002) cells, while Cluster 2 patients had higher scores of progenitor-like (*P* < 0.001) cells [Figure 3D]. Additionally, we used AUCell scoring to evaluate and visualize the activation levels of upregulated genes in each malignant cell across the three clusters, and the results were consistent with the predicted cell clusters [Figure 3E]. These findings were also validated in the Beat AML cohort [Supplementary Figure 3C]. Given the poor prognosis of patients in Cluster 3, we further investigated the malignant evolution of cells in Cluster 3. We found that normal and malignant cells were positioned at opposite ends of the differentiation trajectory and could be well-clustered [Figure 3F and G], suggesting significant transcriptional differences between normal and malignant cells. We then analyzed the gene modules which represent clusters of genes exhibiting synchronized expression patterns along the pseudotime trajectory and performed Gene Ontology (GO) pathway enrichment analysis on the genes within different modules to identify potential driving mechanisms for the formation of malignant cells in Cluster 3. We found that modules 1 and 2, which were highly expressed in normal cells, were enriched in pathways such as variable (diversity) joining [V(D)J] recombination, regulation of cell division, and cytokinesis. In contrast, modules 3 and 4 showed a significant increase in expression in malignant cells as pseudotime progressed. These modules were associated with pathways such as response to metal ion, endoplasmic reticulum stress, calcium ion, fatty acid transport, chronic inflammatory response, interleukin-6 production, and autophagosome formation [Figure 3H].

### Analysis of immune infiltration levels and prediction of immunotherapy efficacy across different clusters

**Figure 4:**
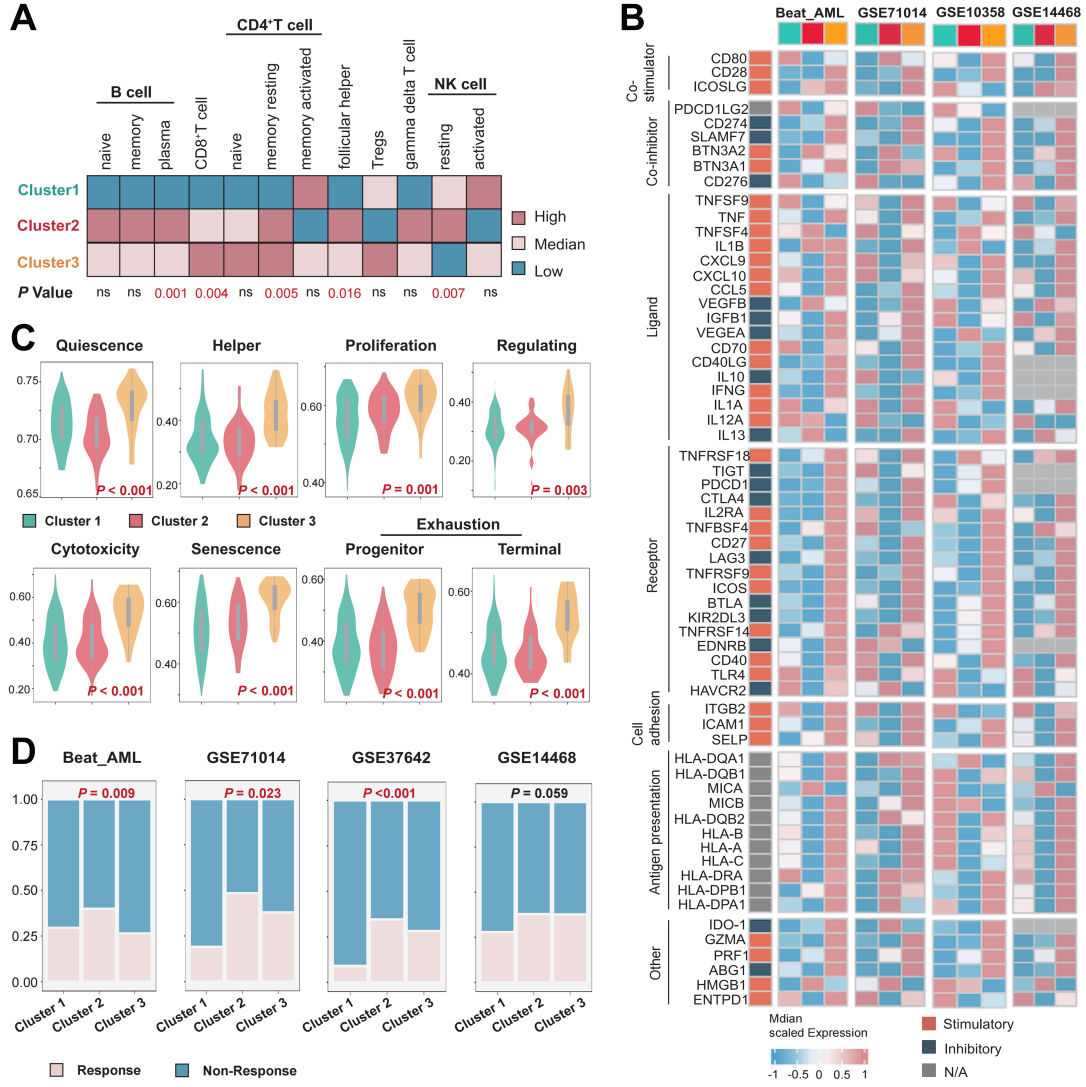
Differences in the immune microenvironment and immune therapy response among the three subtypes. (A) Immune cell infiltration landscape across different clusters; (B) Differences in the expression levels of immune regulatory molecules in multiple cohorts, including Beat-AML, GSE71014, GSE10358, and GSE14468; (C) Differences in T cell states in the TCGA-LAML cohort; (D) Evaluation of immune therapy response across different clusters in the Beat-AML, GSE71014, GSE37642, and GSE14468 cohorts. Differences among three or more groups were assessed with the Kruskal-Wallis test, categorical variables were analyzed using the chi-square test or Fisher’s exact test. *P* < 0.05 was considered statistically significant. ns indicates not significant. AML: Acute myeloid leukemia; TCGA-LAML: The Cancer Genome Atlas-Acute Myeloid Leukemia; NK: natural killer.

We compared the abundance of immune cell infiltration across different clusters using CIBERSORT. We found that in Cluster 2, there was an increase in the infiltration of B cells, follicular helper T cells, and γδ T cells, whereas in Cluster 3, the levels of CD8^+^ T cells, CD4^+^ T cells, and Treg cells were elevated [Figure 4A]. Subsequently, we compared the expression levels of immune checkpoints across clusters. In multiple cohorts, we observed that the mRNA levels of immune checkpoints were higher in Cluster 3 samples compared to the other two clusters [Figure 4B]. The TCellSI tool, which helps evaluate T cell states in patient samples, was consistent with our immune infiltration analysis. We found that T cell scores at various stages were significantly higher in Cluster 3 compared to the other two clusters, indicating high levels of T cell infiltration and exhaustion in Cluster 3 [Figure 4C]. Finally, we predicted the response of AML patients to immunotherapy across multiple cohorts. We discovered that patients in Cluster 2 were more likely to benefit from immunotherapy, while those in Cluster 1 were less likely to gain from it [Figure 4D].

### Cluster 3 patients exhibit resistance to Venetoclax

**Figure 5:**
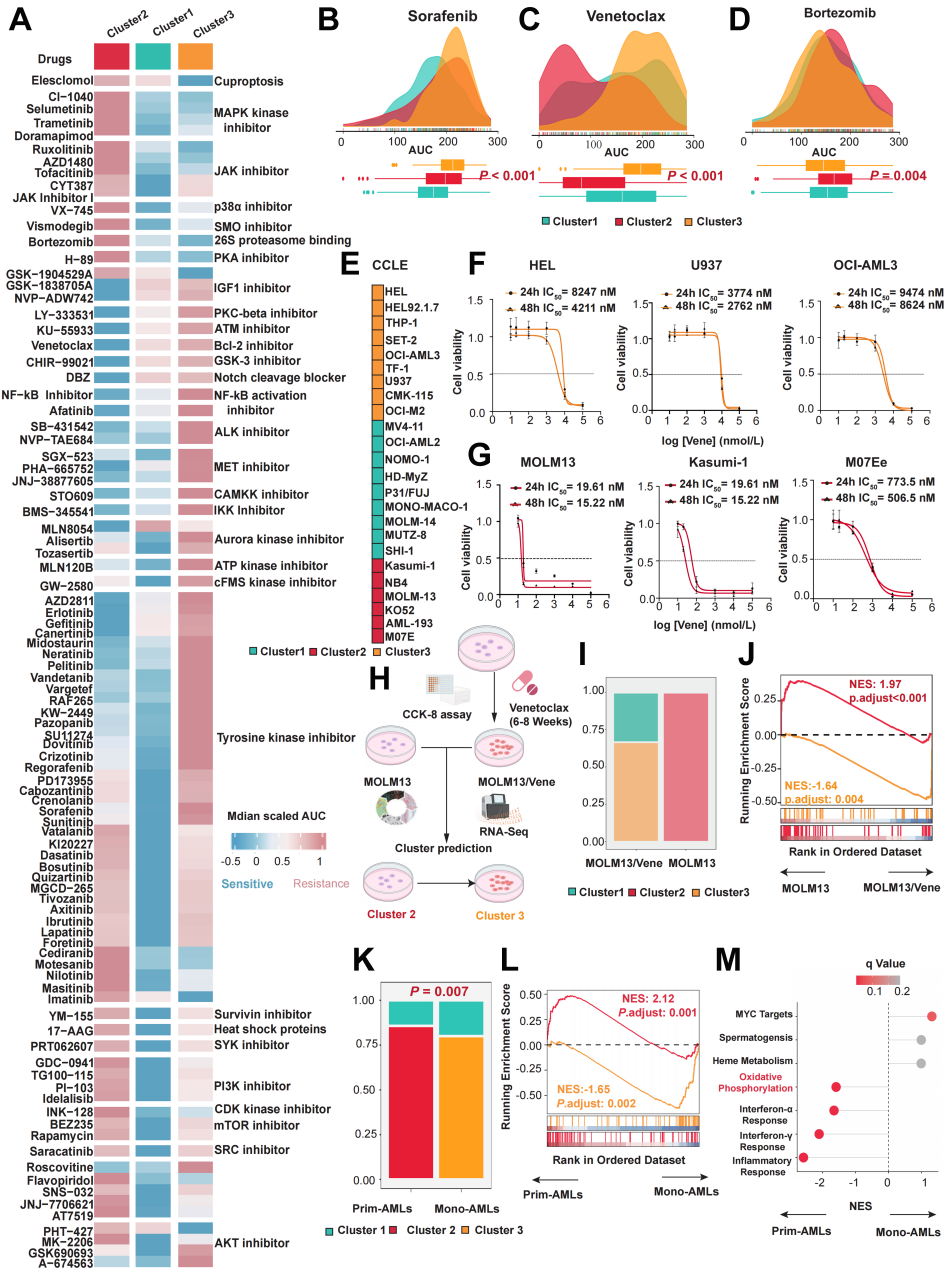
Identification of suitable small molecule treatments for the three clusters. (A) Heatmap showing the median AUC values of drug responses in the three subtypes within the BEAT AML cohort; (B-D) Mountain plots illustrating AUC values for Sorafenib (*P* < 0.001), Venetoclax (*P* < 0.001), and Bortezomib (*P* = 0.004) in the three clusters; (E) Heatmap displaying the distribution of different AML cell lines in the three clusters from the CCLE database; (F) IC_50_ curves of Venetoclax in Cluster 3 AML cell lines (HEL, U937, OCI-AML3) after 24/48 h of treatment; (G) IC_50_ curves of Venetoclax in Cluster 2 AML cell lines (MOLM13, Kasumi-1, M07e) after 24/48 h of treatment; (H) Flowchart for construction and classification prediction of Venetoclax-resistant AML cell lines; (I) Bar chart describing the proportion of clusters in the MOLM13 or MOLM13/Vene; (J) GSEA of DEGs between MOLM13 and MOLM13/Vene in the gene signatures from upregulated genes of Cluster 2 and Cluster 3; (K) The bar chart describes the proportion of clusters in the primary AML cells in the GSE132511 cohort; (L) GSEA of DEGs between mono-AML and prim-AML in the gene signatures from upregulated genes of Cluster 2 and Cluster 3; (M) GSEA of DEGs between mono-AML and prim-AML in the gene signatures from hallmark pathways. Differences among three or more groups were assessed with the Kruskal-Wallis test, categorical variables were analyzed using the chi-square test or Fisher’s exact test. AUC: Area under the curve; AML: acute myeloid leukemia; CCLE: Cancer Cell Line Encyclopedia; IC_50_: half-maximal inhibitory concentration; GSEA: gene set enrichment analysis; DEGs: differentially expressed genes; CCK-8: Cell Counting Kit-8; RNA-Seq: RNA sequencing; NES: normalized enrichment score; MYC: MYC proto-oncogene.

To enable precision medicine in AML, identifying optimal therapeutic strategies for each cluster is essential. We first normalized and visualized the median AUC values of various pathway inhibitors across clusters using drug response data from *ex vivo* samples in the Beat AML cohort [Figure 5A]. The results indicated that Heat shock proteins, CDK kinase inhibitors, Tyrosine kinase inhibitors, and mechanistic target of rapamycin (mTOR) inhibitors were more effective in patients within Cluster 1 [Figure 5B and Supplementary Figure 4A]. Patients in Cluster 2 showed greater sensitivity to B-cell lymphoma 2 (Bcl-2) inhibitors, Notch cleavage blockers, Aurora kinase inhibitors, NF-κB activation inhibitors, and mesenchymal-epithelial transition factor (MET) inhibitors [Figure 5C and Supplementary Figure 4B]. Conversely, Cluster 3 patients exhibited better responses to Elesclomol and 26S proteasome-binding inhibitors, while displaying resistance to Venetoclax [Figure 5D and Supplementary Figure 4C]. Given the marked differential efficacy of Venetoclax across the three clusters, we selected it as a case study to validate the reliability of our drug screening approach. Following our established methodology, we classified AML cell lines from the CCLE database into the respective clusters [Figure 5E and Supplementary Figure 4D]. We then treated three cell lines from Cluster 3 (HEL, U937, OCI-AML3) and three from Cluster 2 (MOLM13, Kasumi-1, M07e) with Venetoclax. The results confirmed that Venetoclax was significantly more effective against Cluster 2-derived cells [Figure 5F and G]. To further investigate the cluster-specific differential effects of Venetoclax, we generated a Venetoclax-resistant MOLM13 cell line (MOLM13/Vene). Transcriptome sequencing of both parental and resistant cells was performed, followed by subtype prediction [Figure 5H, Supplementary Figure 4E and F]. Strikingly, whereas all parental MOLM13 cells were classified into Cluster 2, nearly all MOLM13/Vene cells underwent a transition to Cluster 3 [Figure 5I]. Subsequent DEA between MOLM13 and MOLM13/Vene groups, coupled with pathway enrichment analysis of the deregulated genes, indicated that genes upregulated in MOLM13/Vene were enriched in pathways associated with Cluster 3 marker genes, while those upregulated in the parental MOLM13 were linked to Cluster 2 marker gene pathways [Figure 5J]. Additionally, we obtained bulk transcriptome data from a cohort comprising five mono-AML and seven prim-AML cases. According to prior research by van Galen *et al.*^[23]^, prim-AML samples exhibit progenitor-like characteristics and are more sensitive to Venetoclax, while mono-AML samples tend to resist Venetoclax. We first conducted subtype predictions, finding that nearly all mono-AML samples were classified into Cluster 3 [Figure 5K-L], whereas nearly all prim-AML samples were classified into Cluster 2. Additionally, we found that DEGs in mono-AML were significantly enriched in pathways related to oxidative phosphorylation and interferon response [Figure 5M]. Collectively, these results, spanning cell line models to a clinical cohort, strongly indicate that AML patients belonging to Cluster 3 exhibit pronounced resistance to Venetoclax. To address this resistance, we prioritized Elesclomol and Bortezomib as they exhibited the lowest AUC values among all candidates as illustrated in Supplementary Figure 4G and Supplementary Table 8. These findings provide a robust rationale for investigating these agents as strategies to target Cluster 3 and overcome Venetoclax resistance.

### Elesclomol rescues Venetoclax resistance as a novel therapeutic strategy for Cluster 3 patients

**Figure 6:**
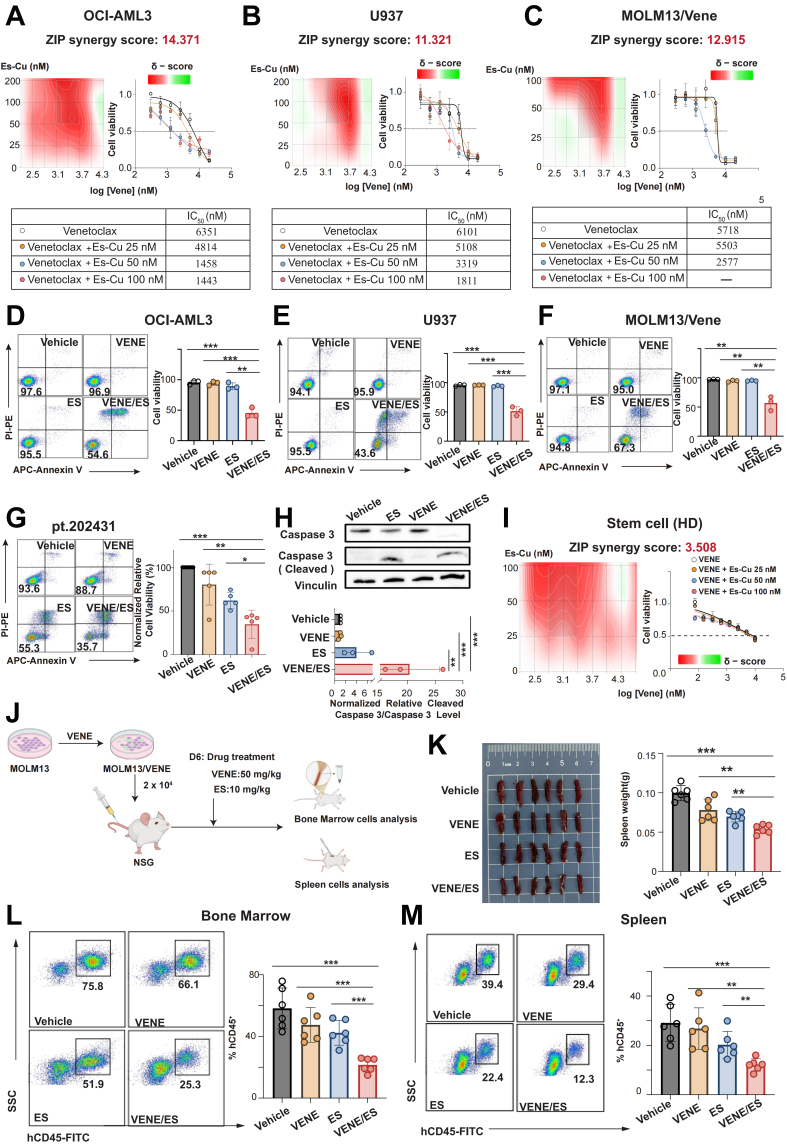
*In vitro* and *in vivo* validation of the synergistic anti-AML effects of Elesclomol and Venetoclax. (A-C) Dose-response curves and matrices for Venetoclax and ES-Cu in OCI-AML3 (ZIP synergy score: 14.371), U937 (ZIP synergy score: 11.321), and MOLM13/Vene (ZIP synergy score: 12.915) cells, with treatment for 24 h before viability measurement using CCK-8; (D-F) Apoptosis of the AML cell lines in Cluster 3 (U937, OCI-AML3,MOLM13/VENE) detected by flow cytometry after treatment with 100 nM ES-Cu, 3 μM Venetoclax, and a combination of ES-Cu and Venetoclax; (G) Apoptosis of primary cells from refractory AML patients detected by flow cytometry after treatment with 100 nM ES-Cu, 1 μM Venetoclax, and a combination of ES-Cu and Venetoclax; (H) Expression levels of apoptotic protein caspase-3 and its cleaved form in primary AML patient samples following treatment with ES-Cu, Venetoclax, and their combination; (I) Dose-response curves and matrices for Venetoclax and ES-Cu in stem cell from healthy donor (ZIP synergy score: 3.058); (J) Establishment of the Venetoclax-resistant mouse model and the corresponding dosing regimen; (K) Spleen morphology and weight in mice after combination treatment with Elesclomol (10 mg/kg) and Venetoclax (50 mg/kg); (L and M) Tumor burden (hCD45^+^ cells) in the bone marrow (L) and spleen (M) of mice following combination treatment with Elesclomol and Venetoclax. Results are expressed as mean ± SD of at least three independent experiments. Statistical significance was determined using an unpaired Student’s *t*-test. *P* < 0.05 was considered statistically significant. * indicates *P* < 0.05, ** indicates *P* < 0.01, *** indicates *P* < 0.001. AML: Acute myeloid leukemia; ES-Cu: elesclomol-copper; ZIP: zero interaction potency; CCK-8: Cell Counting Kit-8; SD: standard deviation; IC_50_: half-maximal inhibitory concentration; APC: allophycocyanin; SSC: side scatter.

Given the observed resistance of Cluster 3 to Venetoclax, we hypothesized, based on our prior drug screen, that the cuprotosis activator Elesclomol could offer a viable treatment strategy for this cluster. To test this, we treated three cell lines in Cluster 3 (OCI-AML3, U937, and MOLM13/Vene) with increasing concentrations of elesclomol-copper (ES-Cu). Elesclomol demonstrated significant cytotoxicity in all tested lines [Supplementary Figure 5A and B]. Moreover, ES-Cu effectively targeted primary cells from newly diagnosed and relapsed/refractory AML patients [Supplementary Figure 5C]. We subsequently investigated whether Elesclomol and Venetoclax could act synergistically to inhibit AML cell growth. The aforementioned Cluster 3 cell lines were treated with different concentrations of Elesclomol, Venetoclax, or their combination for 24 h. The combination treatment demonstrated significantly enhanced inhibitory effects compared to monotherapies across all three cell lines (ZIP synergy score > 10) [Figure 6A-C]. Further investigation revealed that while both Venetoclax and Elesclomol monotherapies induced moderate apoptosis, their combination substantially augmented this apoptotic effect within 24 h [Figure 6D-F]. This finding was consistently validated in primary AML patient samples [Figure 6G and H, Supplementary Figure 5D]. Notably, this enhanced apoptotic effect was specific to AML cells, as no combined toxicity was observed in HSCs from healthy donors, indicating a favorable safety profile for this combination strategy [Figure 6I]. Finally, we evaluated the therapeutic efficacy of the Elesclomol-Venetoclax combination in a MOLM13/Vene xenograft model [Figure 6J]. Mice receiving the combined treatment showed significantly reduced spleen size compared to those receiving single-agent treatments [Figure 6K]. Flow cytometric analysis of human cluster of differentiation 45 positive (hCD45^+^) leukemic cells in the bone marrow and spleen further demonstrated that the combination treatment significantly reduced AML burden compared to either monotherapy or vehicle control groups [Figure 6L and M].

### Bortezomib sensitizes Cluster 3 patients to Venetoclax as a novel combination strategy

**Figure 7:**
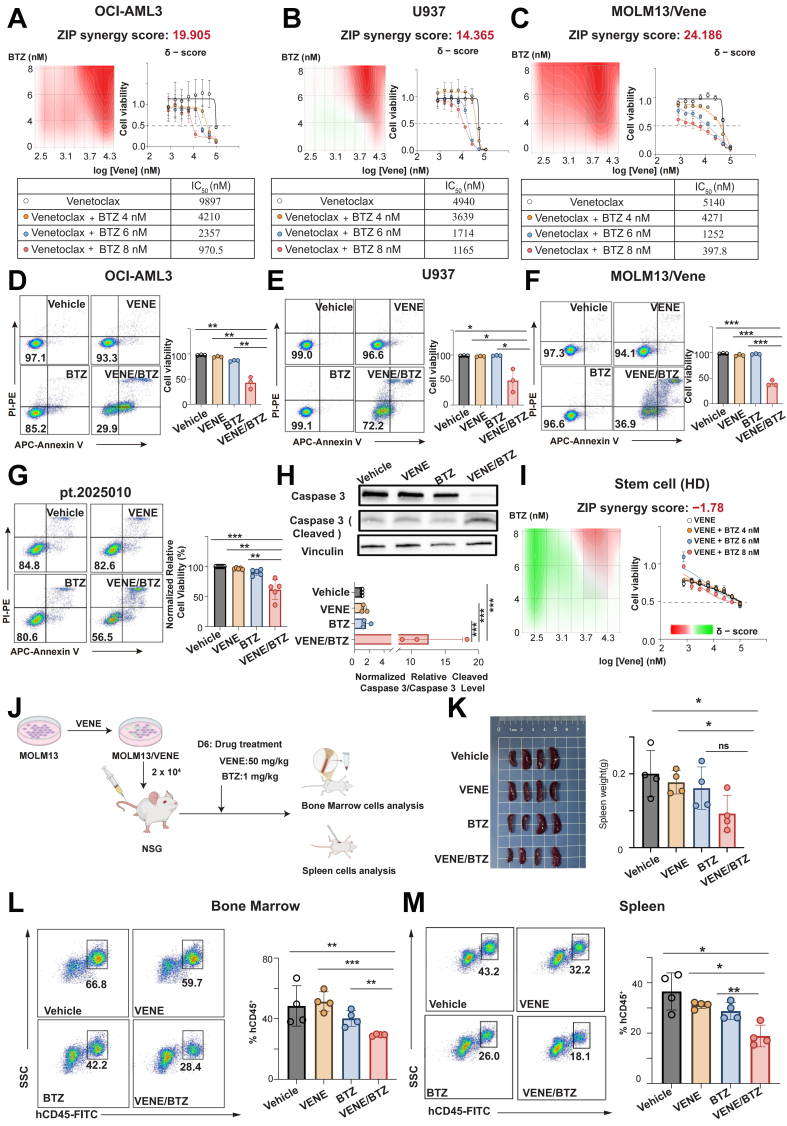
*In vitro* and *in vivo* validation of the synergistic anti-AML effects of Bortezomib and Venetoclax. (A-C) Dose-response curves and matrices for Venetoclax and Bortezomib in OCI-AML3 (ZIP synergy score: 19.905), U937 (ZIP synergy score: 14.365), and MOLM13/Vene (ZIP synergy score: 24.186) cells, with treatment for 24 h before viability measurement using CCK-8; (D-F) Apoptosis of the AML cell lines in Cluster 3 (U937, OCI-AML3, MOLM13/VENE) detected by flow cytometry after treatment with 6 nM Bortezomib, 3 μM Venetoclax, and a combination of Bortezomib and Venetoclax; (G) Apoptosis of primary cells from refractory AML patients detected by flow cytometry after treatment with 8 nM Bortezomib, 1 μM Venetoclax, and a combination of Bortezomib and Venetoclax; (H) Expression levels of apoptotic protein caspase-3 and its cleaved form in primary AML patient samples following treatment with Bortezomib, Venetoclax, and their combination; (I) Dose-response curves and matrices for Venetoclax and Bortezomib in stem cell from healthy donor (ZIP synergy score: -1.78); (J) Establishment of the Venetoclax-resistant mouse model and the corresponding dosing regimen; (K) Spleen morphology and weight in mice after combination treatment with Bortezomib (1 mg/kg) and Venetoclax (50 mg/kg); (L and M) Tumor burden (hCD45^+^ cells) in the bone marrow (L) and spleen (M) of mice following combination treatment with Bortezomib and Venetoclax. Results are expressed as mean ± SD of at least three independent experiments. Statistical significance was determined using an unpaired Student’s *t*-test. *P* < 0.05 was considered statistically significant. ns indicates not significant, * indicates *P* < 0.05, ** indicates *P* < 0.01, *** indicates *P* < 0.001. AML: Acute myeloid leukemia; ZIP: zero interaction potency; CCK-8: Cell Counting Kit-8; SD: standard deviation; BTZ: Bortezomib; IC_50_: half-maximal inhibitory concentration; APC: allophycocyanin; SSC: side scatter.

**Figure 8:**
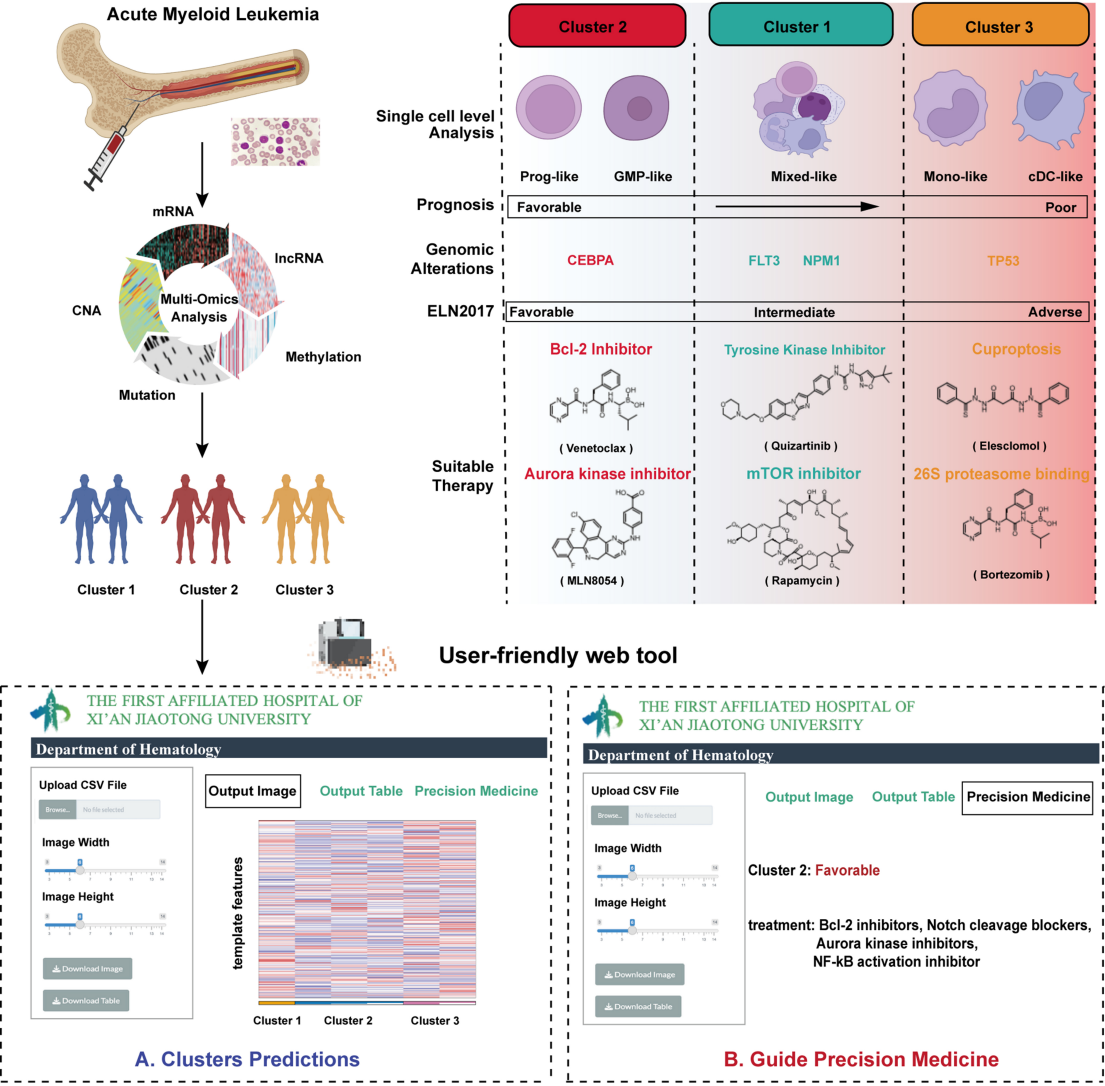
Schematic overview of the study and development of a user-friendly web tool. Created in BioRender. Yang, R. (2026) https://BioRender.com/9csio13. mRNA: Messenger RNA; CNA: copy number alteration; lncRNA: long non-coding RNA; GMP: granulocyte-macrophage progenitor; cDC: conventional dendritic cell; CEBPA: CCAAT/enhancer-binding protein alpha; FLT3: FMS-like tyrosine kinase 3; NPM1: nucleophosmin 1; TP53: tumor protein 53; ELN: European LeukemiaNet; Bcl-2: B-cell lymphoma 2; mTOR: mechanistic target of rapamycin.

Parallel to our findings with Elesclomol, our drug sensitivity screen also identified the proteasome inhibitor Bortezomib as a candidate agent with selective activity against Cluster 3. To validate this, we exposed three representative Cluster 3 cell lines (OCI-AML3, U937, and MOLM13/Vene) to increasing concentrations of Bortezomib. The treatment elicited potent cytotoxic effects in all tested lines [Supplementary Figure 6A]. Given the single-agent efficacy of Bortezomib and the pressing need to overcome Venetoclax resistance in this cluster, we subsequently explored whether Bortezomib could enhance Venetoclax efficacy in these resistant models. Combination treatment of Bortezomib and Venetoclax for 24 h yielded superior growth inhibition compared to single-agent treatments, achieving ZIP synergy scores above 10 in all three cell lines [Figure 7A-C]. This combinatorial effect extended to apoptosis induction, where the drug combination generated significantly enhanced cell death compared to individual treatments [Figure 7D-F]. The synergistic activity was maintained in primary AML specimens [Figure 7G and H, Supplementary Figure 6B] while sparing healthy donor HSCs, indicating a favorable therapeutic window [Figure 7I]. The therapeutic potential of this combination was further substantiated *in vivo* using the MOLM13/Vene xenograft model [Figure 7J]. Combination-treated mice showed remarkable reduction in spleen size [Figure 7K] alongside significantly decreased hCD45^+^ leukemic cell burden in both bone marrow [Figure 7L] and spleen compared to monotherapy groups [Figure 7M], confirming the dual regimen’s ability to overcome Venetoclax resistance in Cluster 3 patients. Finally, we developed a user-friendly web tool that allows users to upload patient transcriptomic data for multi-omics subtype prediction [Figure 8].

## DISCUSSION

A major challenge in AML precision medicine is predicting and overcoming resistance to targeted drugs such as Venetoclax. Our study directly addresses this unmet need. By integrating five dimensions of multi-omics data and applying nine complementary clustering algorithms with consensus, we established a robust molecular classification system that categorizes AML patients into three subtypes with distinct prognoses and biological features [Figure 8]. The prognostic value of this classification was consistently validated across multiple independent cohorts. While the feature selection was initially guided by survival-associated features to ensure clinical utility, the consistent performance across these diverse, independent datasets indicates that our framework captures intrinsic biological patterns rather than being overfit to the discovery cohort. This validation across platforms mitigates potential methodological bias and confirms the generalizability of the identified subtypes. Crucially, the stability of the classification is further supported by the high degree of concordance among the various data layers, which suggests that the subtypes represent shared biological states rather than artifacts of any single platform. Furthermore, our classification aligns well with established cytogenetic and molecular markers, such as the association of biallelic CEBPA mutations with favorable outcomes^[28]^ and TP53 mutations with poor prognosis^[29]^, underscoring its clinical relevance. Although the distribution of favorable risk patients showed minor variations between Cluster 1 and Cluster 2 across different cohorts, the primary clinical utility of our framework is the stable identification of Cluster 3 as a high-risk biological entity with consistent therapeutic resistance.

Among the subtypes identified, Cluster 3 emerges as a high-risk entity with distinct biological behaviors and pronounced therapeutic resistance. Our multi-omics analysis reveals that Cluster 3 is characterized not only by a high frequency of TP53 mutations and globally elevated DNA methylation but also, as evidenced by single-cell transcriptomics^[30]^, by its association with monocytic and dendritic cell differentiation states. This finding resonates with previous work by Pei *et al.*, who identified that monocytic AML possesses a unique transcriptional profile, loses BCL2 expression, and relies on myeloid cell leukemia 1 (MCL1)-mediated oxidative phosphorylation for survival, leading to Venetoclax resistance^[19,31]^. Rodriguez-Sevilla *et al.* further discovered that in myelodysplastic syndromes (MDS) patients who initially responded to Venetoclax but eventually relapsed, HSCs displayed expansion with a granulo-monocytic-biased transcriptional differentiation state^[32]^. In a real-world study, Zhao *et al.* observed that newly diagnosed AML patients with the French-American-British (FAB) monocytic subtype exhibited a lower complete remission (CR) rate compared to non-monocytic subtypes^[33]^. These findings suggest that monocytic-like AML has reduced sensitivity to venetoclax treatment, which is highly consistent with our finding in Cluster 3. Our pseudotime analysis further delineated the evolutionary trajectory of Cluster 3 malignant cells, uncovering the activation of key pathways including endoplasmic reticulum stress, autophagy, fatty acid transport, and chronic inflammatory response. These insights are congruent with findings from Sheth *et al.* and Stevens *et al.*, who reported that enhanced mitochondrial metabolism and fatty acid oxidation contribute to Venetoclax resistance in AML cells^[34,35]^. Notably, the significant upregulation of inflammatory mediators such as S100A8 and S100A9 during malignant evolution - proteins previously linked to poor prognosis and Venetoclax resistance in AML by Karjalainen *et al.* - suggests their potential role as key effector molecules in the pathogenesis of Cluster 3^[36]^.

Beyond cell-intrinsic metabolic and stress pathways, Cluster 3 is defined by a unique immune microenvironment. Although this subtype exhibits increased infiltration of immune cells, including CD8^+^ T cells and Tregs, and elevated expression of immune checkpoint molecules, T cell state analysis revealed a functionally exhausted phenotype. This paradoxical state of “immune-rich but suppressive” microenvironment may be driven by persistent interferon-gamma (IFN-γ) signaling, which is indicated by our pathway enrichment analyses. While early studies suggested IFN-γ could enhance anti-tumor immunity^[37]^, recent evidence is consistent with the idea that prolonged activation of the IFN-γ pathway creates a chronic inflammatory environment. This environment appears to reduce cytotoxic T cell activity and is associated with the progression of CD8^+^ T cells toward an exhausted phenotype, suggesting a potential means for tumor cells to evade the immune system^[38,39]^. Xie *et al.* further demonstrated that low doses of IFN-γ can preserve the self-renewal capacity of AML stem cells^[40]^. Interestingly, the IFN-γ signaling score has been closely associated with venetoclax resistance in primary AML cells^[41]^. Our previous research also found that high expression of the IFN-γ pathway-stimulated gene 2′-5′-oligoadenylate synthetase 1 (*OAS1*) is correlated with poor prognosis in AML patients^[42]^. Consequently, the immune-infiltrated yet suppressed microenvironment in Cluster 3 may not only form the basis for immune evasion but also indirectly foster therapy resistance.

The ultimate goal of our study was to transcend molecular classification and explore effective therapeutic strategies for the identified high-risk subgroup. Guided by the distinct biological features of Cluster 3, we conducted a multi-omics-informed drug sensitivity screen in the Beat AML cohort. The results indicated that while Cluster 3 exhibits profound resistance to Venetoclax, it demonstrates marked sensitivity to both Elesclomol and Bortezomib. This finding aligns with a mechanistic rationale, as Elesclomol is consistent with the induction of intense oxidative stress^[43]^ and cuprotosis^[44]^. Such effects potentially target cancer cells with hyperactive mitochondrial metabolism^[45-47]^, which appears to be a core vulnerability of Cluster 3. Bortezomib, a proteasome inhibitor, is likely effective against Cluster 3 through mechanisms such as inhibiting NF-κB signaling and disrupting the clearance of misfolded proteins, thereby synergizing with the observed endoplasmic reticulum stress^[14]^. Subsequent preclinical experiments robustly validated the translational potential of these findings. *In vitro*, the combination of either Elesclomol or Bortezomib with Venetoclax exhibited powerful synergistic lethality in Cluster 3-derived cell lines, and primary patient samples, significantly augmenting apoptosis induction. Furthermore, in a Venetoclax-resistant xenograft model *in vivo*, both combination regimens significantly reduced leukemic burden. The compelling efficacy and safety data presented here, particularly for the Venetoclax-Bortezomib combination, provide strong justification for its prompt clinical evaluation^[48]^. The well-established use of Bortezomib in hematological malignancies provides a clear and rapid path for clinical translation^[49]^, as its safety profile and dosing protocols are already familiar to oncologists. Our findings therefore not only provide a robust preclinical rationale but also lay the groundwork for initiating clinical trials of Venetoclax combined with Bortezomib in patients with relapsed/refractory AML, addressing a critical unmet need in this high-risk population.

In conclusion, this study establishes a novel multi-omics classification system that identifies a distinct AML subtype (Cluster 3) characterized by Venetoclax resistance and poor prognosis. It provides a detailed analysis of its unique molecular, cellular, and microenvironmental features. More importantly, using this biological understanding, we systematically identified and preclinically validated two promising combination strategies to overcome this resistance. The Venetoclax-Bortezomib combination stands out as a strong candidate for rapid clinical translation. To support clinical application, we developed an interactive web platform. Overall, our work delivers a comprehensive precision medicine package: a robust diagnostic classifier combined with multiple therapeutic options for this challenging AML subset.

Although this study involved comprehensive multi-omics integration and extensive preclinical validation, several limitations warrant discussion. First, while our classification system demonstrated robust performance across multiple retrospective cohorts, its clinical utility requires validation through well-designed prospective clinical trials to establish its predictive value in guiding treatment decisions. Second, although our drug sensitivity screen identified Elesclomol and Bortezomib as promising candidates for overcoming Venetoclax resistance in Cluster 3, this selection was primarily constrained by the drugs’ AUC values and clinical feasibility. A broader range of compounds, including those targeting additional pathways identified in our multi-omics analysis (such as IFN-γ signaling, fatty acid metabolism, or endoplasmic reticulum stress pathways), warrants further investigation. Third, while the mechanistic insights into how Elesclomol and Bortezomib synergize with Venetoclax are supported by existing literature, deeper investigation is required to fully elucidate the molecular basis of these synergistic effects. Finally, although our *in vivo* studies demonstrated significant efficacy of the combination regimens in xenograft models, future research should diversify the types of mouse models to provide more comprehensive preclinical evidence for the feasibility of these drug combinations from multiple perspectives. Addressing these limitations will not only facilitate the clinical application of the novel AML subtypes identified in this study and the clinical translation of the combination regimens, but also further demonstrate the value of multi-omics in advancing precision therapy for AML and overcoming targeted drug resistance.

## DECLARATIONS

### Availability of data and materials

The data and scripts used in this study are available on GitHub (https://github.com/RunyuYang0701/aml_multiomics_subtypes_xjtu). AML microarray and single-cell data were obtained from GEO (http://www.ncbi.nlm.nih.gov/geo/). AML patient RNA-seq data were acquired from TCGA (https://portal.gdc.cancer.gov/) and Beat AML (https://biodev.github.io/BeatAML2/). A user-friendly web tool for cluster prediction is available at https://runyuyang0701.shinyapps.io/mluti-omics_subtypes_aml_xjtu/.

### AI and AI-assisted tools statement

During the preparation of this manuscript, the AI tool Gemini 3 Flash (Version 3.0, released 2025-12-06) was used solely for language editing. The tool did not influence the study design, data collection, analysis, interpretation, or the scientific content of the work. All authors take full responsibility for the accuracy, integrity, and final content of the manuscript.

### Copyright

© The Author(s) 2026.

## References

[1] Döhner H, Weisdorf DJ, Bloomfield CD (2015). Acute myeloid leukemia. N Engl J Med.

[2] Vardiman JW, Thiele J, Arber DA (2009). The 2008 revision of the World Health Organization (WHO) classification of myeloid neoplasms and acute leukemia: rationale and important changes. Blood.

[3] Zhang Z, Huang J, Zhang Z (2024). Application of omics in the diagnosis, prognosis, and treatment of acute myeloid leukemia. Biomark Res.

[4] Shimony S, Stahl M, Stone RM (2023). Acute myeloid leukemia: 2023 update on diagnosis, risk-stratification, and management. Am J Hematol.

[5] Pereira MP, Herrity E, Kim DDH (2024). TP53-mutated acute myeloid leukemia and myelodysplastic syndrome: biology, treatment challenges, and upcoming approaches. Ann Hematol.

[6] Negotei C, Colita A, Mitu I (2023). A review of FLT3 kinase inhibitors in AML. J Clin Med.

[7] Ediriwickrema A, Gentles AJ, Majeti R (2023). Single-cell genomics in AML: extending the frontiers of AML research. Blood.

[8] Karczewski KJ, Snyder MP (2018). Integrative omics for health and disease. Nat Rev Genet.

[9] Bottomly D, Long N, Schultz AR (2022). Integrative analysis of drug response and clinical outcome in acute myeloid leukemia. Cancer Cell.

[10] Zeng AGX, Bansal S, Jin L (2022). A cellular hierarchy framework for understanding heterogeneity and predicting drug response in acute myeloid leukemia. Nat Med.

[11] Severens JF, Karakaslar EO, van der Reijden BA (2024). Mapping AML heterogeneity - multi-cohort transcriptomic analysis identifies novel clusters and divergent ex-vivo drug responses. Leukemia.

[12] Alharbi F, Vakanski A (2023). Machine learning methods for cancer classification using gene expression data: a review. Bioengineering.

[13] Addala V, Newell F, Pearson JV (2024). Computational immunogenomic approaches to predict response to cancer immunotherapies. Nat Rev Clin Oncol.

[14] Sogbein O, Paul P, Umar M, Chaari A, Batuman V, Upadhyay R (2024). Bortezomib in cancer therapy: mechanisms, side effects, and future proteasome inhibitors. Life Sci.

[15] Tomasson MH, Xiang Z, Walgren R (2008). Somatic mutations and germline sequence variants in the expressed tyrosine kinase genes of patients with de novo acute myeloid leukemia. Blood.

[16] Wang YH, Lin CC, Hsu CL (2021). Distinct clinical and biological characteristics of acute myeloid leukemia with higher expression of long noncoding RNA KIAA0125. Ann Hematol.

[17] Taskesen E, Bullinger L, Corbacioglu A (2011). Prognostic impact, concurrent genetic mutations, and gene expression features of AML with CEBPA mutations in a cohort of 1182 cytogenetically normal AML patients: further evidence for CEBPA double mutant AML as a distinctive disease entity. Blood.

[18] Herold T, Jurinovic V, Batcha AMN (2018). A 29-gene and cytogenetic score for the prediction of resistance to induction treatment in acute myeloid leukemia. Haematologica.

[19] Pei S, Pollyea DA, Gustafson A (2020). Monocytic subclones confer resistance to Venetoclax-based therapy in patients with acute myeloid leukemia. Cancer Discov.

[20] Lu X, Meng J, Zhou Y, Jiang L, Yan F (2021). MOVICS: an R package for multi-omics integration and visualization in cancer subtyping. Bioinformatics.

[21] Szklarczyk D, Kirsch R, Koutrouli M (2023). The STRING database in 2023: protein-protein association networks and functional enrichment analyses for any sequenced genome of interest. Nucleic Acids Res.

[22] Yu G, Wang LG, Han Y, He QY (2012). clusterProfiler: an R package for comparing biological themes among gene clusters. OMICS.

[23] van Galen P, Hovestadt V, Wadsworth Ii MH (2019). Single-cell RNA-seq reveals AML hierarchies relevant to disease progression and immunity. Cell.

[24] Qiu X, Hill A, Packer J, Lin D, Ma YA, Trapnell C (2017). Single-cell mRNA quantification and differential analysis with Census. Nat Methods.

[25] Yang JM, Zhang N, Luo T (2024). TCellSI: a novel method for T cell state assessment and its applications in immune environment prediction. Imeta.

[26] Chen Y, He LN, Zhang Y (2024). tigeR: tumor immunotherapy gene expression data analysis R package. iMeta.

[27] Ianevski A, Giri AK, Aittokallio T (2022). SynergyFinder 3.0: an interactive analysis and consensus interpretation of multi-drug synergies across multiple samples. Nucleic Acids Res.

[28] Döhner H, Estey E, Grimwade D (2017). Diagnosis and management of AML in adults: 2017 ELN recommendations from an international expert panel. Blood.

[29] Aldoss I, Zhang J, Pillai R (2019). Venetoclax and hypomethylating agents in TP53-mutated acute myeloid leukaemia. Br J Haematol.

[30] Li K, Du Y, Cai Y (2023). Single-cell analysis reveals the chemotherapy-induced cellular reprogramming and novel therapeutic targets in relapsed/refractory acute myeloid leukemia. Leukemia.

[31] Pei S, Shelton IT, Gillen AE (2023). A novel type of monocytic leukemia stem cell revealed by the clinical use of Venetoclax-based therapy. Cancer Discov.

[32] Rodriguez-Sevilla JJ, Ganan-Gomez I, Ma F (2024). Hematopoietic stem cells with granulo-monocytic differentiation state overcome venetoclax sensitivity in patients with myelodysplastic syndromes. Nat Commun.

[33] Zhao L, Yang J, Chen M (2024). Myelomonocytic and monocytic acute myeloid leukemia demonstrate comparable poor outcomes with venetoclax-based treatment: a monocentric real-world study. Ann Hematol.

[34] Sheth AI, Althoff MJ, Tolison H (2024). Targeting acute myeloid leukemia stem cells through perturbation of mitochondrial calcium. Cancer Discov.

[35] Stevens BM, Jones CL, Pollyea DA (2020). Fatty acid metabolism underlies venetoclax resistance in acute myeloid leukemia stem cells. Nat Cancer.

[36] Karjalainen R, Liu M, Kumar A (2019). Elevated expression of S100A8 and S100A9 correlates with resistance to the BCL-2 inhibitor venetoclax in AML. Leukemia.

[37] Mittal D, Gubin MM, Schreiber RD, Smyth MJ (2014). New insights into cancer immunoediting and its three component phases - elimination, equilibrium and escape. Curr Opin Immunol.

[38] Qiu J, Xu B, Ye D (2023). Cancer cells resistant to immune checkpoint blockade acquire interferon-associated epigenetic memory to sustain T cell dysfunction. Nat Cancer.

[39] Vadakekolathu J, Minden MD, Hood T (2020). Immune landscapes predict chemotherapy resistance and immunotherapy response in acute myeloid leukemia. Sci Transl Med.

[40] Xie X, Zhang W, Zhou X (2023). Low doses of IFN-γ maintain self-renewal of leukemia stem cells in acute myeloid leukemia. Oncogene.

[41] Wang B, Reville PK, Yassouf MY (2024). Comprehensive characterization of IFNγ signaling in acute myeloid leukemia reveals prognostic and therapeutic strategies. Nat Commun.

[42] Yang R, Du Y, Zhang M (2023). Multi-omics analysis reveals interferon-stimulated gene OAS1 as a prognostic and immunological biomarker in pan-cancer. Front Immunol.

[43] Nagai M, Vo NH, Shin Ogawa L (2012). The oncology drug elesclomol selectively transports copper to the mitochondria to induce oxidative stress in cancer cells. Free Radical Biol Med.

[44] Tsvetkov P, Coy S, Petrova B (2022). Copper induces cell death by targeting lipoylated TCA cycle proteins. Science.

[45] Cierlitza M, Chauvistré H, Bogeski I (2015). Mitochondrial oxidative stress as a novel therapeutic target to overcome intrinsic drug resistance in melanoma cell subpopulations. Exp Dermatol.

[46] Corazao-Rozas P, Guerreschi P, Jendoubi M (2013). Mitochondrial oxidative stress is the Achille’s heel of melanoma cells resistant to Braf-mutant inhibitor. Oncotarget.

[47] Tsvetkov P, Detappe A, Cai K (2019). Mitochondrial metabolism promotes adaptation to proteotoxic stress. Nat Chem Biol.

[48] Ohlstrom D, Bakhtiari M, Mumme H (2025). Longitudinal single-cell analysis reveals treatment-resistant stem and mast cells with potential treatments for pediatric AML. Leukemia.

[49] Kumar SK, Harrison SJ, Cavo M (2025). Venetoclax or placebo in combination with bortezomib and dexamethasone in relapsed or refractory multiple myeloma (BELLINI): final overall survival results from a randomised, phase 3 study. Lancet Haematol.

