# Supplementary material for: Multi-omics classification of acute myeloid leukemia guides drug combinations to overcome Venetoclax resistance
Source: Cancer Drug Resist. 2026 Mar 26;9:10. doi: 10.20517/cdr.2025.228 (PMC13103256; doi:10.20517/cdr.2025.228)
Supplement: Supplementary file 1 [file cdr-9-10-SupplementaryMaterials.pdf]

Supplementary Materials

Multi-omics classification of acute myeloid leukemia guides drug combinations to overcome Venetoclax resistance

Runyu Yang[1,#], Hui Feng[1,#], Mengyao Zhang[1,#], Yi Liu[1], Minna Luo[1], Ruimin Liu[1], Kaiyao Wang[2], Qijing Li[1], Wenjuan Wang[1], Jing Chen[1], Yue Du[1], Jiayi Xiao[1], Bingyu Yang[1], Fan Niu[1], Pengcheng He[1]

[1]Department of Hematology, The First Affiliated Hospital of Xi'an Jiaotong University, Xi'an 710061, Shaanxi, China.
[2]Shaanxi University of Chinese Medicine, Xi'an-Xianyang New Economic Zone, Xi'an 712046, Shaanxi, China.
[#]These authors contributed equally to this work and share first authorship.

Correspondence to: Dr. Bingyu Yang, Prof. Fan Niu, Prof. Pengcheng He, Department of Hematology, The First Affiliated Hospital of Xi'an Jiaotong University, Xi'an 710061, Shaanxi, China. E-mail: byyang@xjtu.edu.cn; niufan@xjtufh.edu.cn; hepengcheng@xjtu.edu.cn

Supplementary Figure 1 (related to Figure 1)

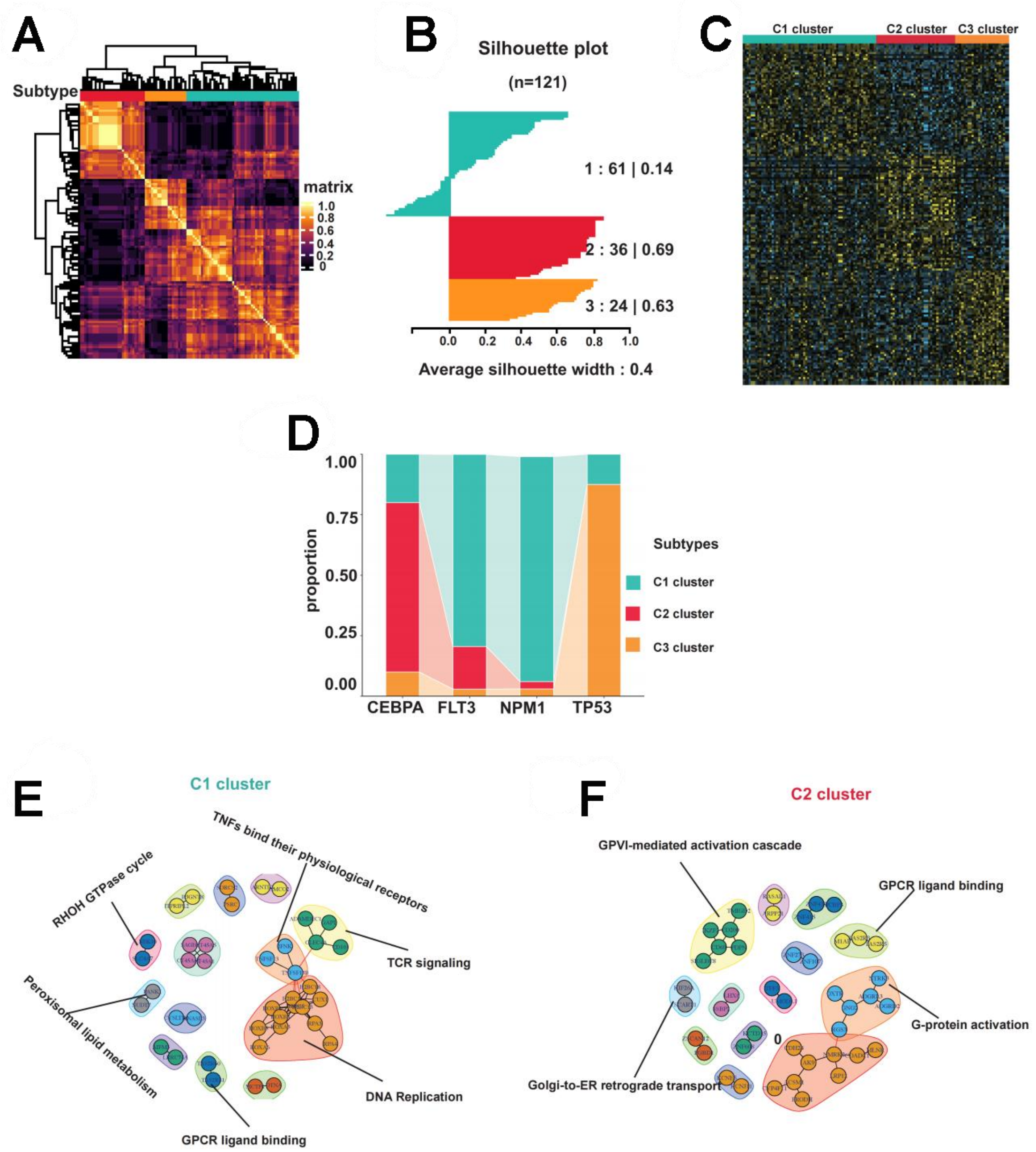

Supplementary Figure 1. Identification of three distinct clusters and evaluation of different clinical indicators. (A) Consistency clustering of heatmap display. (B) Silhouette scores of different clusters. (C) Different biomarker genes among different clusters. (D) Comparison of CEBPA, FLT3, NPM1, and TP53 gene mutations with the three clusters. (E) PPI network interactions of upregulated differential genes in Cluster 1, showing enriched pathways for different gene modules. (F) PPI network interactions of upregulated differential genes in Cluster 2, showing enriched pathways for different gene modules.

Supplementary Figure 2 (related to Figure 2)

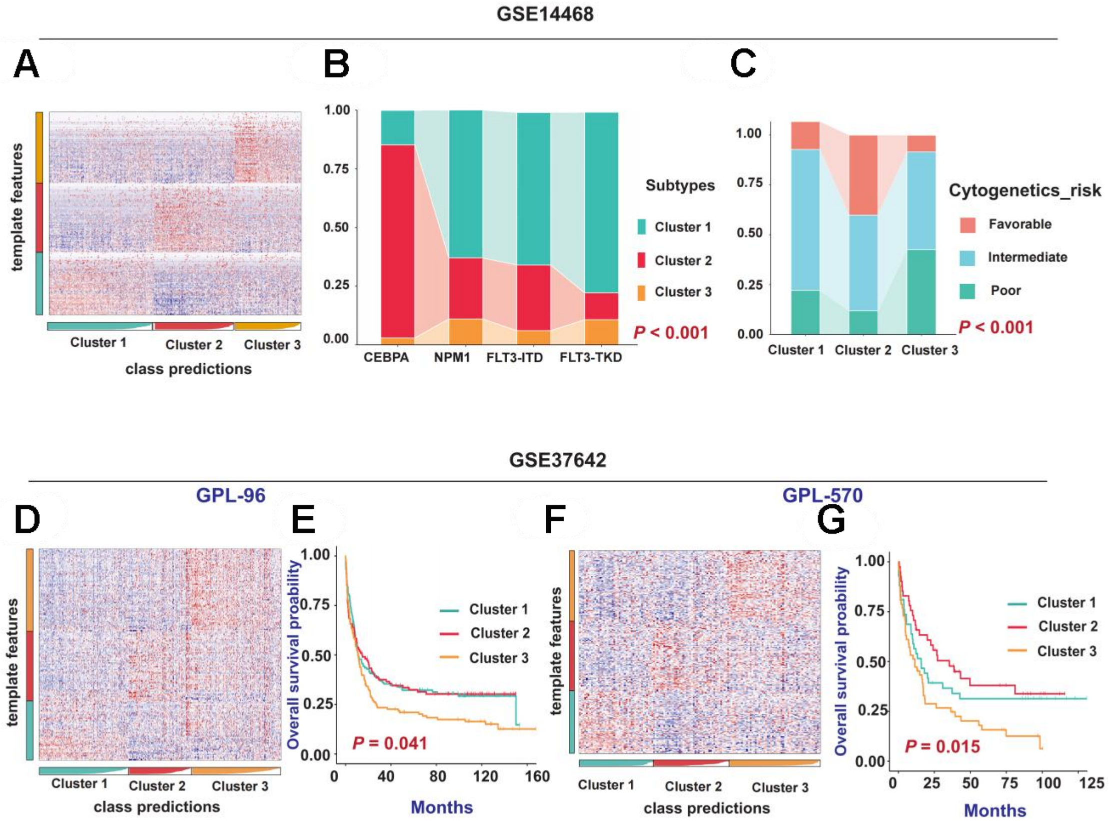

Supplementary Figure 2. Reproduction of AML molecular subtyping results in external cohorts. (A) Prediction and classification of patient samples using NTP in the GSE14468 cohort. (B) Comparison of CEBPA, FLT3, NPM1,gene mutations with the three clusters. (C) Comparison of the existing AML cytogenetic risk standards with the three subtypes identified through clustering in this study. (D) Prediction and classification of patient samples using NTP in the GSE37642-GPL96 cohort. (E) Kaplan-Meier survival curves illustrate differences in overall survival among patients with different clusters in the GSE37642-GPL96 cohort (*P* = 0.041). (F) Prediction and classification of patient samples using NTP in the GSE37642-GPL570 cohort. (G) Kaplan-Meier survival curves illustrate differences in overall survival among patients with different clusters in the GSE37642-GPL96 cohort (*P* = 0.015).

Supplementary Figure 3 (related to Figure 3)

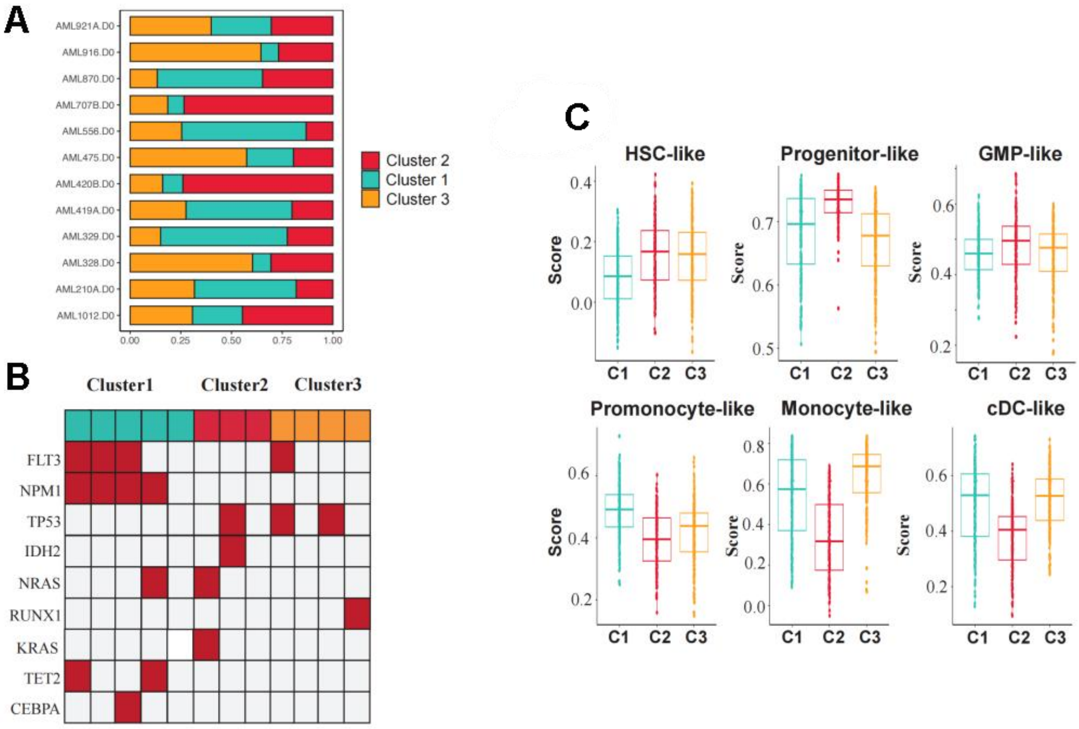

Supplementary Figure 3. Single-cell depiction of three AML subtypes. (A) Proportion of clusters in the different patients. (B) Comparison of CEBPA, FLT3, NPM1, TP53,IDH1,NRAS,RUNX1,CEBPA and TET2 gene mutations with the different patients . (C) Boxplot of different AML cell types scores: HSC-like (p <0.001) , progenitor-like (p <0.001) , GMP-like (p <0.001) , promonocyte-like (p <0.001) , monocyte-like (p <0.001) , cDC-like (p <0.001) in patients of different clusters from the BEAT-AML cohort .

Supplementary Figure 4 (related to Figure 5)

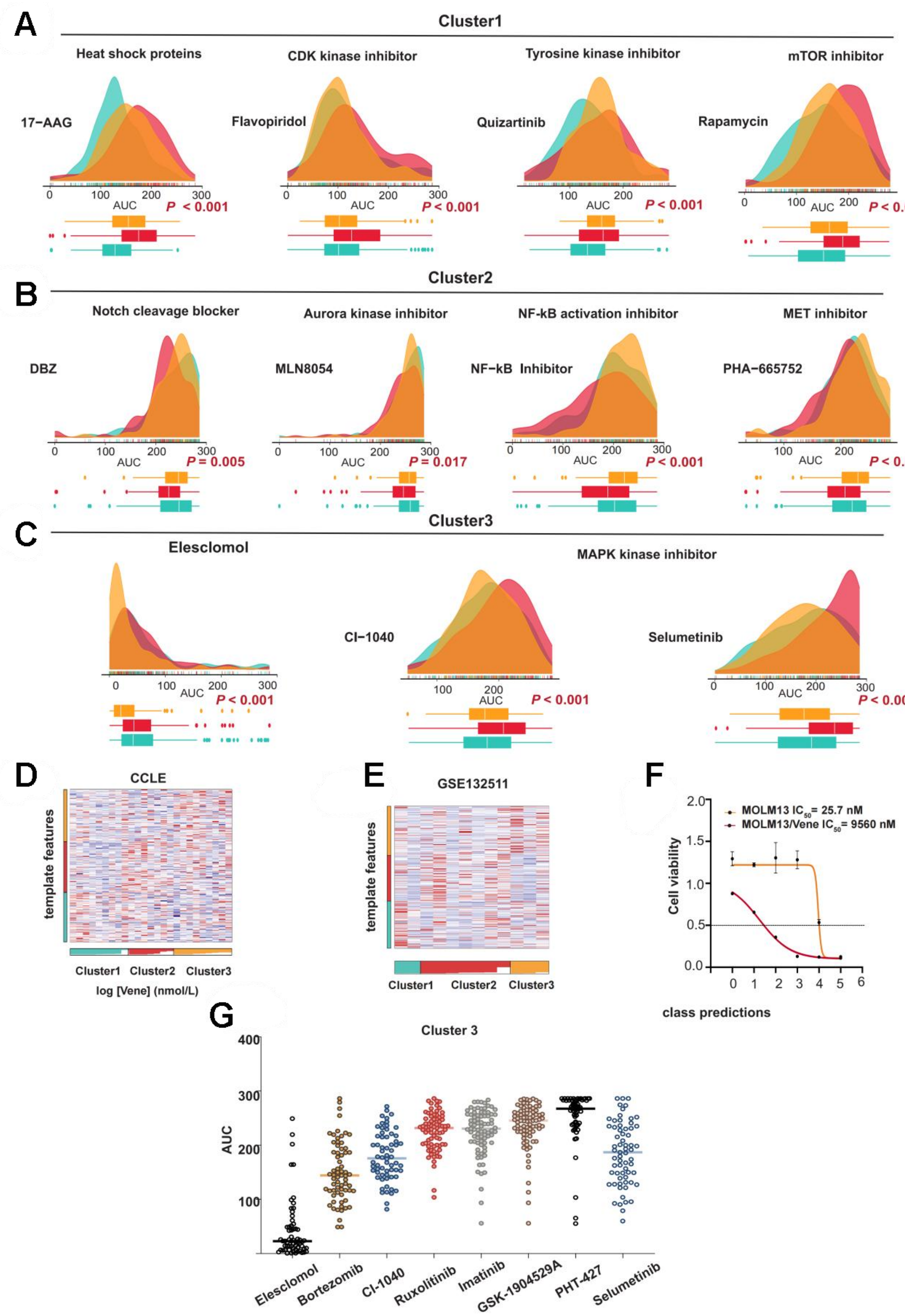

Supplementary Figure 4. Identification of suitable small molecule treatments for the three clusters. (A-C) Mountain plots illustrating AUC values for suitable therapy strageties for Cluster 1 , Cluster 2 and Cluster 3. (D) Prediction and classification of patient samples using NTP in the CCLE. (E) Prediction and classification of patient samples using NTP in the MOLM13&MOLM13/Vene cohort. (F) IC_{50} curves of Venetoclax in MOLM-13 or MOLM-13/Vene after 24 hours of treatment. (G) AUC of candidate drug in cluster 3 patients.

Supplementary Figure 5 (related to Figure 6)

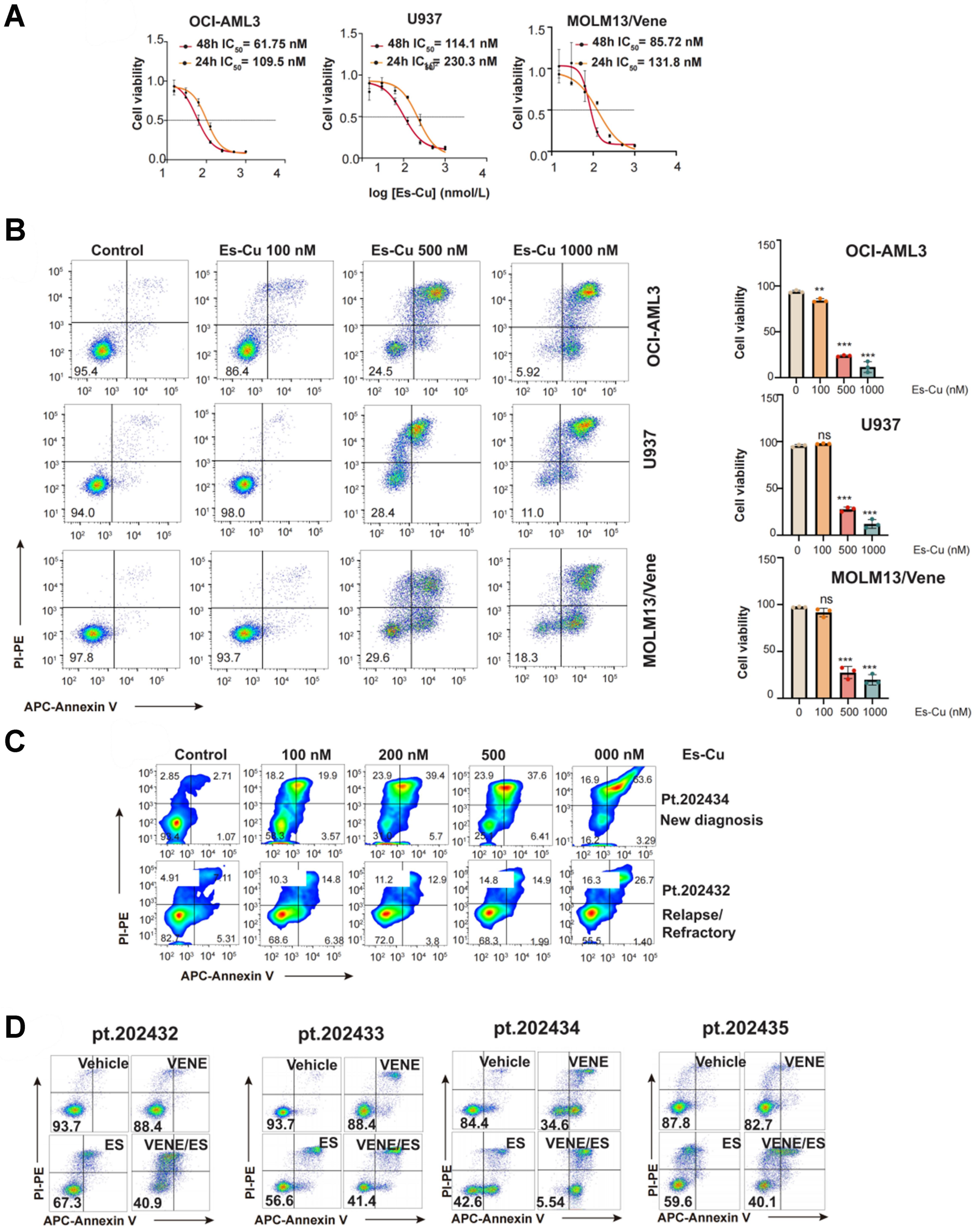

Supplementary Figure 5. Treating AML cells with different concentrations of ES-CU (A) IC_{50} curves of Elesclomol (with 100 nM $CuCl_2$, ES-Cu) in U937, OCI-AML3, and MOLM13/Vene cell lines after 24/48 hours of treatment. (B) Apoptosis of OCI-AML3 ,U937 ,MOLM13/Vene detected by flow cytometry after treatment with different concentrations of ES-Cu. (C) Apoptosis of primary cells from newly diagnosed/refractory AML patients detected by flow cytometry after treatment with different concentrations of ES-Cu. (D) Apoptosis of primary cells from newly diagnosed/refractory AML patients detected by flow cytometry after treatment with 100 nM ES-Cu, 1 μM Venetoclax, and a combination of ES-Cu and Venetoclax.

Supplementary Figure 6 (related to Figure 7)

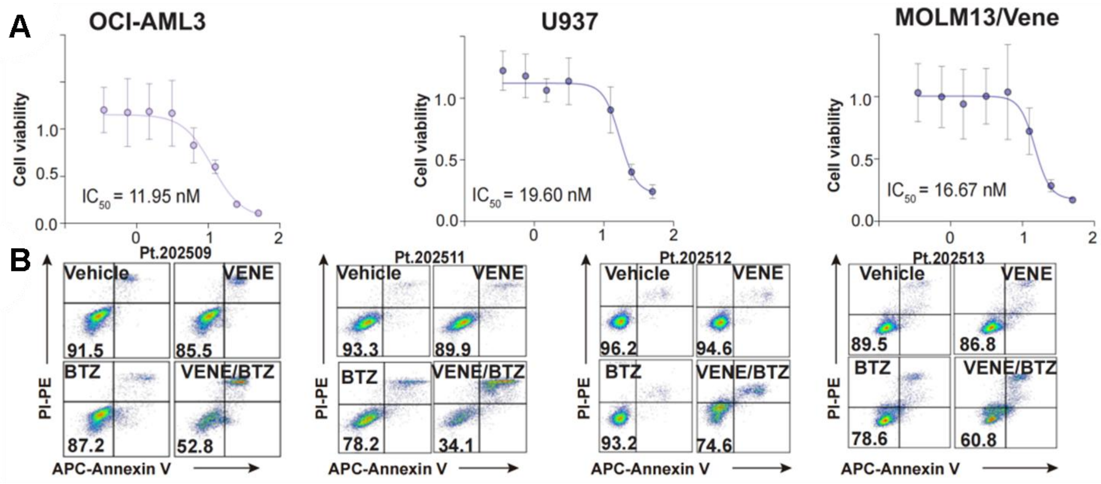

Supplementary Figure 6. Treating AML cells with different concentrations of Bortezomib. (A) IC_{50} curves of Bortezomib in U937, OCI-AML3, and MOLM13/Vene cell lines after 24 hours of treatment. (B) Apoptosis of primary cells from newly diagnosed/refractory AML patients detected by flow cytometry after treatment with 6 nM Bortezomib, 3 μM Venetoclax, and a combination of Bortezomib and Venetoclax.

Supplementary Table 1. Basic information of Multi-omics Datasets

Dataset	Description	Ref. (PMID)
TCGA-LAML	RNA seq data of AML Patients	-
BeatAML2	RNA seq data of AML Patients & Drug response data	35868306
GSE10358	Microarrays data of AML Patients	19651600
GSE71014	Microarrays data of AML Patients	33225420
GSE14468	Microarrays data of AML Patients	19171880
GSE37642	Microarrays data of AML Patients	24923295
GSE132511	RNA seq data of primitive and monocytic primary AML	31974170
GSE116256	Single cell-RNA seq data AML Patients & HD samples	30827681

Supplementary Table 2. Selection of Survival related Genes

Gene	HR	Z	pValue	lower	upper
TCTA	1.01E+00	4.712044	2.45E-06	1.01E+00	1.01E+00
SFXN3	1.00E+00	4.526002	6.01E-06	1.00E+00	1.01E+00
KCTD17	1.02E+00	4.483777	7.33E-06	1.01E+00	1.04E+00
TCF15	1.11E+00	4.424873	9.65E-06	1.06E+00	1.16E+00
KBTBD8	1.02E+00	4.410263	1.03E-05	1.01E+00	1.03E+00
DEFB103B	5.62E+01	4.350472	1.36E-05	9.15E+00	3.45E+02
OR5M10	9.52E+00	4.299509	1.71E-05	3.41E+00	2.66E+01
PARP3	1.01E+00	4.187627	2.82E-05	1.01E+00	1.02E+00
CSK	1.00E+00	4.11056	3.95E-05	1.00E+00	1.00E+00
LRRC37B	9.85E-01	-4.009067	6.10E-05	9.78E-01	9.92E-01
ZFYVE19	1.02E+00	3.978987	6.92E-05	1.01E+00	1.03E+00
RBMY1A1	6.49E+01	3.93828	8.21E-05	8.13E+00	5.17E+02
ZDHHC11	1.06E+00	3.906496	9.36E-05	1.03E+00	1.09E+00
DNAH3	1.10E+00	3.869032	1.09E-04	1.05E+00	1.16E+00
ZNF124	9.89E-01	-3.848606	1.19E-04	9.83E-01	9.94E-01
FNDC3A	9.94E-01	-3.759582	1.70E-04	9.91E-01	9.97E-01
GALNT1	9.96E-01	-3.752854	1.75E-04	9.94E-01	9.98E-01
NFYA	9.94E-01	-3.71858	2.00E-04	9.92E-01	9.97E-01
MTCH1	1.00E+00	3.698928	2.17E-04	1.00E+00	1.00E+00
MTX1	1.02E+00	3.678259	2.35E-04	1.01E+00	1.04E+00
FBXW12	4.77E-01	-3.665642	2.47E-04	3.21E-01	7.09E-01
RBMY1E	3.60E+01	3.647155	2.65E-04	5.25E+00	2.47E+02
2-Mar	1.01E+00	3.647032	2.65E-04	1.00E+00	1.01E+00
SLC29A4	9.13E-01	-3.630679	2.83E-04	8.70E-01	9.59E-01
ABHD11	1.02E+00	3.565435	3.63E-04	1.01E+00	1.02E+00
OAZ1	1.00E+00	3.529028	4.17E-04	1.00E+00	1.00E+00
IGSF5	1.42E+00	3.524791	4.24E-04	1.17E+00	1.72E+00
MAP7D1	1.00E+00	3.515877	4.38E-04	1.00E+00	1.00E+00
AP2M1	1.00E+00	3.508639	4.50E-04	1.00E+00	1.00E+00
TUBGCP2	1.01E+00	3.505055	4.57E-04	1.00E+00	1.01E+00
LBX1	1.89E+00	3.504683	4.57E-04	1.32E+00	2.69E+00

LILRB4	1.00E+00	3.490323	4.82E-04	1.00E+00	1.01E+00
ARHGAP27	1.00E+00	3.484066	4.94E-04	1.00E+00	1.01E+00
KCNE1B	1.07E+00	3.457618	5.45E-04	1.03E+00	1.12E+00
OTUB1	1.00E+00	3.450002	5.61E-04	1.00E+00	1.01E+00
BLOC1S1	1.01E+00	3.432945	5.97E-04	1.01E+00	1.02E+00
TRNAU1AP	1.02E+00	3.429082	6.06E-04	1.01E+00	1.04E+00
RABGGTA	1.01E+00	3.425522	6.14E-04	1.00E+00	1.01E+00
CTAGE8	2.89E+00	3.41613	6.35E-04	1.57E+00	5.32E+00
HELZ2	1.01E+00	3.402889	6.67E-04	1.00E+00	1.01E+00
NOTCH4	1.04E+00	3.330808	8.66E-04	1.02E+00	1.07E+00
S100A5	1.10E+00	3.312998	9.23E-04	1.04E+00	1.16E+00
DPP3	1.01E+00	3.31024	9.32E-04	1.00E+00	1.01E+00
B3GALT5	1.05E+00	3.294558	9.86E-04	1.02E+00	1.08E+00

Supplementary Table 3. Selection of Survival related lncRNAs

Gene	HR	Z	pValue	lower	upper
LINC01679	1.018881	5.946759	2.74E-09	1.0126191	1.0251817
AC133961.1	1.0071536	5.63413	1.76E-08	1.0046593	1.0096542
AC084048.1	1.0497161	5.051398	4.39E-07	1.0301391	1.0696651
TRAF3IP2-AS1	0.9934886	-4.643055	3.43E-06	0.9907527	0.9962321
AL050402.1	1.5195578	4.540493	5.61E-06	1.2684594	1.8203625
TRERNA1	1.0224423	4.455696	8.36E-06	1.012509	1.032473
HCG24	1.0323962	4.398796	1.09E-05	1.0178338	1.0471669
AC010247.2	1.0020291	4.328749	1.50E-05	1.0011099	1.0029492
LINC01700	1.0221633	4.328428	1.50E-05	1.0120673	1.03236
TSPEAR-AS2	1.0048159	4.320938	1.55E-05	1.0026286	1.0070081
AC121764.2	1.2594832	4.318015	1.57E-05	1.1342654	1.3985246
LINC02256	1.0018448	4.2102	2.55E-05	1.0009856	1.0027048
AC036103.1	0.9929235	-4.201384	2.65E-05	0.9896395	0.9962185
AL033527.3	1.0293916	4.189601	2.79E-05	1.0155357	1.0434366
RHPN1-AS1	1.0041909	4.122719	3.74E-05	1.0021964	1.0061895
LINC02145	1.0116357	4.058261	4.94E-05	1.0059993	1.0173036
AL844908.2	1.0175661	4.04079	5.33E-05	1.0090075	1.0261972
AC004832.6	1.0214645	3.97379	7.07E-05	1.0108207	1.0322203
GRM7-AS1	1.0187464	3.969152	7.21E-05	1.0094459	1.0281325
LINC01011	1.0082425	3.948149	7.88E-05	1.0041422	1.0123595
AC025423.1	1.0013495	3.927877	8.57E-05	1.0006759	1.0020236
AC008074.3	1.0024503	3.890234	1.00E-04	1.0012151	1.0036871
AC000120.1	0.9940899	-3.868918	1.09E-04	0.9911093	0.9970796
AC005096.1	0.998208	-3.86787	1.10E-04	0.9973012	0.9991157
TSPEAR-AS1	1.0050082	3.865685	1.11E-04	1.0024659	1.0075571
AC008551.1	1.0026024	3.860866	1.13E-04	1.0012805	1.0039261
AC092436.4	1.0018765	3.856416	1.15E-04	1.0009224	1.0028316
AC084882.1	1.3127266	3.85474	1.16E-04	1.1431097	1.5075116
AL353719.1	1.0088203	3.833476	1.26E-04	1.004301	1.01336
AL353622.1	1.002358	3.815448	1.36E-04	1.001146	1.0035714
AC138150.2	1.015881	3.804128	1.42E-04	1.0076676	1.0241615

AC092468.1	1.136399	3.752647	1.75E-04	1.0629864	1.2148815
AC078851.1	1.0744529	3.73377	1.89E-04	1.0347042	1.1157285
LINC01327	1.0414843	3.731341	1.90E-04	1.0194837	1.0639598
AC131211.1	1.0176902	3.71548	2.03E-04	1.0083197	1.0271478
AC243960.3	1.0031618	3.704629	2.12E-04	1.0014878	1.0048385
LINC00449	1.0438398	3.671678	2.41E-04	1.0202039	1.0680233
AC092287.1	1.0093333	3.664214	2.48E-04	1.0043302	1.0143613
AC105105.1	1.2325925	3.649044	2.63E-04	1.1016377	1.3791142
AL139039.3	1.0843033	3.625261	2.89E-04	1.0378793	1.1328039
AC008770.4	0.9983376	-3.623456	2.91E-04	0.9974396	0.9992365
AL121832.2	1.0050274	3.620822	2.94E-04	1.0023029	1.0077593
AC073517.1	0.9967578	-3.613752	3.02E-04	0.9950037	0.9985149
AC012555.1	1.1504942	3.602561	3.15E-04	1.0660079	1.2416764
AC243562.3	0.8030488	-3.602104	3.16E-04	0.7127061	0.9048434
AL451069.3	1.0024365	3.599415	3.19E-04	1.001109	1.0037658
AL355353.1	1.0039412	3.596598	3.22E-04	1.0017915	1.0060955
AL391704.1	1.0941701	3.587938	3.33E-04	1.0416796	1.1493055
AP002748.3	1.0100821	3.571251	3.55E-04	1.0045364	1.0156585
AL450326.1	1.0052326	3.55897	3.72E-04	1.0023476	1.008126
AC104667.2	1.0566087	3.554016	3.79E-04	1.0250051	1.0891868
AC012456.2	1.0304714	3.543476	3.95E-04	1.0135041	1.0477227
AC239802.1	1.1770654	3.541639	3.98E-04	1.0755216	1.2881963
AC091808.1	1.018629	3.535701	4.07E-04	1.0082598	1.0291049
NADK2-AS1	0.9978303	-3.535442	4.07E-04	0.9966296	0.9990326
AC027243.1	1.0512271	3.527621	4.19E-04	1.0224494	1.0808147
SFTPD-AS1	1.0627955	3.522238	4.28E-04	1.0273813	1.0994304
AC022182.1	1.0057767	3.519592	4.32E-04	1.0025557	1.0090081
AC010260.1	0.9978836	-3.503593	4.59E-04	0.9967016	0.999067
AL391121.1	1.0008975	3.500549	4.64E-04	1.0003949	1.0014003
AC008572.1	1.4159862	3.498953	4.67E-04	1.165312	1.7205837
AC104667.1	1.0213871	3.492213	4.79E-04	1.0093282	1.0335902
AP000777.3	1.2815808	3.491083	4.81E-04	1.1149496	1.4731154
AC080013.5	1.0139335	3.489126	4.85E-04	1.0060828	1.0218454

AC130352.1	0.9915188	-3.483725	4.94E-04	0.9867789	0.9962815
AC015712.1	1.0137304	3.46508	5.30E-04	1.0059411	1.0215801
DSCR10	1.0546251	3.456422	5.47E-04	1.0232938	1.0869158
AF064858.2	1.0019944	3.452241	5.56E-04	1.0008616	1.0031284
AL049647.1	0.998294	-3.424117	6.17E-04	0.9973187	0.9992701
AC084819.1	1.0247234	3.416655	6.34E-04	1.010467	1.0391809
BX470102.1	1.0053485	3.411497	6.46E-04	1.0022722	1.0084342
AL365181.3	1.003591	3.399148	6.76E-04	1.0015188	1.0056674
AC096677.1	1.0024018	3.395942	6.84E-04	1.0010149	1.0037906
AC008278.1	1.0579284	3.379958	7.25E-04	1.0239402	1.0930447
AL355376.1	2.0172568	3.379296	7.27E-04	1.3427717	3.0305411
AC022182.2	1.0015666	3.366203	7.62E-04	1.0006542	1.0024799
CFAP58-AS1	1.0063569	3.364885	7.66E-04	1.0026493	1.0100783
AL121899.1	1.0402726	3.36428	7.67E-04	1.0166175	1.0644782
AP003068.2	1.0025017	3.352099	8.02E-04	1.0010382	1.0039673
AC018953.1	1.3231665	3.344279	8.25E-04	1.1228986	1.559152
FO393418.1	1.0051405	3.336919	8.47E-04	1.002118	1.0081722
AL512413.1	0.995948	-3.335447	8.52E-04	0.9935746	0.998327
AL022313.2	1.0081434	3.335242	8.52E-04	1.0033499	1.0129598
AL080317.3	0.9929729	-3.334187	8.55E-04	0.9888651	0.9970977
AC026358.1	0.8396295	-3.319401	9.02E-04	0.7572943	0.9309163
AC093520.1	1.0121673	3.311842	9.27E-04	1.0049489	1.0194376
AC002454.1	0.9997188	-3.309152	9.36E-04	0.9995522	0.9998853
AL591848.4	0.9986156	-3.305207	9.49E-04	0.9977955	0.9994363
AL121612.2	1.188419	3.304928	9.50E-04	1.0727769	1.316527
AC011471.2	0.9889688	-3.299385	9.69E-04	0.9824735	0.995507

Supplementary Table 4. Selection of Survival related copy number alterations

Gene	HR	Z	pValue	lower	upper
chr10.109576298-109911045	1753.083771	4.497803579	6.87E-06	67.65227832	45427.92621
chr6.170559546-170572998	11455.74295	4.277372883	1.89E-05	158.1651271	829728.0762
chr1.100706880-103051417	665.3881053	4.153133935	3.28E-05	30.95978212	14300.53122
chr1.103051417-103181721	665.3881053	4.153133935	3.28E-05	30.95978212	14300.53122
chr1.103181721-103373589	665.3881053	4.153133935	3.28E-05	30.95978212	14300.53122
chr1.107101018-107144758	662.6447884	4.147468519	3.36E-05	30.76321152	14273.48101
chr1.107144758-107839920	662.6447884	4.147468519	3.36E-05	30.76321152	14273.48101
chr1.107839920-108061800	662.6447884	4.147468519	3.36E-05	30.76321152	14273.48101
chr1.108061800-108074669	662.6447884	4.147468519	3.36E-05	30.76321152	14273.48101
chr1.108074669-108082416	662.6447884	4.147468519	3.36E-05	30.76321152	14273.48101
chr1.103373589-103502567	653.0557643	4.128756947	3.65E-05	30.10675971	14165.65035
chr1.105015208-105524223	649.8879109	4.123835422	3.73E-05	29.91996851	14116.1344
chr1.105524223-105821916	649.8879109	4.123835422	3.73E-05	29.91996851	14116.1344
chr1.105821916-107101018	649.8879109	4.123835422	3.73E-05	29.91996851	14116.1344
chr1.103502567-104978076	645.9500362	4.117713892	3.83E-05	29.68865533	14054.23872
chr1.104978076-105015208	645.9500362	4.117713892	3.83E-05	29.68865533	14054.23872
chr12.88794587-90040945	612789.5418	3.97361643	7.08E-05	856.5496287	438399609.2
chr12.95586374-95699929	1052793.1	3.96882544	7.22E-05	1117.558634	991780904.1
chr12.95699929-95700071	1052793.1	3.96882544	7.22E-05	1117.558634	991780904.1
chr12.95700071-95960628	1052793.1	3.96882544	7.22E-05	1117.558634	991780904.1
chr12.90040945-91253865	575530.2498	3.947585855	7.89E-05	794.7168087	416796354.1
chr14.39421087-39887211	155775.9595	3.945320799	7.97E-05	410.1664883	59161706.92
chr14.36487078-37357277	154064.7467	3.937646501	8.23E-05	403.2026627	58868525.39
chr14.37357277-39421087	153469.7043	3.935714814	8.29E-05	401.2475123	58699305.11
chr14.39887211-41303154	152690.7144	3.935665805	8.30E-05	400.1941718	58257855.61
chr14.104970556-10598803	126606.887	3.924987634	8.67E-05	358.5233375	44709234.15
chr14.33126543-33918760	145577.9221	3.903913686	9.46E-05	372.3556648	56915829.17
chr14.36485443-36487078	141555.9594	3.890446557	0.00010006	359.7058256	55706881.03
chr14.33918760-35039207	141367.6268	3.889736925	0.000100353	359.076679	55656095.44
chr14.35039207-35101594	141367.6268	3.889736925	0.000100353	359.076679	55656095.44
chr14.35101594-36485443	141367.6268	3.889736925	0.000100353	359.076679	55656095.44
chr14.104303490-104743850	120166.2185	3.886613626	0.000101652	329.7010776	43797006.01
chr14.104743850-104970556	120166.2185	3.886613626	0.000101652	329.7010776	43797006.01
chr14.44597628-45261492	129388.8877	3.88299868	0.000103176	340.1262273	49221385.79
chr14.45261492-46246423	129388.8877	3.88299868	0.000103176	340.1262273	49221385.79
chr14.46246423-46592900	129388.8877	3.88299868	0.000103176	340.1262273	49221385.79
chr14.41303154-42271141	129063.6603	3.88084897	0.000104092	338.5866638	49196941.85
chr14.42271141-42921942	129063.6603	3.88084897	0.000104092	338.5866638	49196941.85
chr14.42921942-42952536	129063.6603	3.88084897	0.000104092	338.5866638	49196941.85

chr14.42952536-44597628	129063.6603	3.88084897	0.000104092	338.5866638	49196941.85
chr14.30038802-31551249	107236.0062	3.874781373	0.00010672	306.1012341	37567836.2
chr14.47679344-48401820	122884.5592	3.856584191	0.000114983	318.3842447	47428901.21
chr14.48401820-48415444	122884.5592	3.856584191	0.000114983	318.3842447	47428901.21
chr14.27669798-27939115	105659.2668	3.855631696	0.000115431	295.1648324	37822529.74
chr12.80339843-80862086	274255.0865	3.854924107	0.000115766	471.2233034	159618278.5
chr14.57238411-57397690	122840.4378	3.85251833	0.000116909	316.3334885	47702104.61
chr14.57397690-57547575	122840.4378	3.85251833	0.000116909	316.3334885	47702104.61
chr14.57547575-58455758	122828.3969	3.852421663	0.000116955	316.2709391	47702185.75
chr12.84863708-84919096	266893.6236	3.852302371	0.000117012	462.9568573	153863594.8
chr12.84919096-85650557	266893.6236	3.852302371	0.000117012	462.9568573	153863594.8
chr12.85650557-85809907	266893.6236	3.852302371	0.000117012	462.9568573	153863594.8
chr12.84252853-84468344	266891.7178	3.852286783	0.00011702	462.9433254	153865894.8
chr14.29731662-29733309	102553.3018	3.850368136	0.00011794	288.5436444	36449181.68
chr14.29733309-30038802	102553.3018	3.850368136	0.00011794	288.5436444	36449181.68
chr12.80862086-80862862	268977.2612	3.84759773	0.000119282	461.1282206	156895119
chr14.32963166-33126543	131259.7368	3.847170688	0.00011949	324.0925037	53161113.89
chr5.19586552-19945200	1035716.715	3.846509783	0.000119812	891.6905303	1203006062
chr5.19945200-21181358	1035716.715	3.846509783	0.000119812	891.6905303	1203006062
chr5.21181358-23845886	1035716.715	3.846509783	0.000119812	891.6905303	1203006062
chr5.23845886-23876317	1035716.715	3.846509783	0.000119812	891.6905303	1203006062
chr5.23876317-25839780	1035716.715	3.846509783	0.000119812	891.6905303	1203006062
chr5.25839780-26357934	1035716.715	3.846509783	0.000119812	891.6905303	1203006062
chr5.26357934-26560056	1035716.715	3.846509783	0.000119812	891.6905303	1203006062
chr5.26560056-27645758	1035716.715	3.846509783	0.000119812	891.6905303	1203006062
chr12.88723350-88794587	471807.9609	3.845203481	0.000120452	604.9967109	367940433.3
chr14.47478753-47679344	119702.1106	3.844912189	0.000120596	308.6832054	46418447.85
chr14.31551249-32290611	130314.6548	3.841684091	0.000122193	320.1900974	53036959.54
chr14.32290611-32963166	130314.6548	3.841684091	0.000122193	320.1900974	53036959.54
chr12.83676241-84252853	257657.5731	3.838424128	0.000123826	444.706318	149283746
chr14.56047948-56267456	118878.2109	3.832510076	0.000126842	301.766266	46831043.18
chr14.46592900-47132829	117239.6272	3.832172907	0.000127016	299.56939	45882959.52
chr14.47136641-47151941	115732.3833	3.828543992	0.000128904	296.0038373	45249361.15
chr14.47151941-47478753	115732.3833	3.828543992	0.000128904	296.0038373	45249361.15
chr12.81793463-81799667	254893.574	3.828047813	0.000129164	434.8086623	149423734.4
chr14.52536512-54224586	115828.4405	3.827025824	0.000129701	295.4233537	45413564.87
chr14.54224586-56047948	115828.4405	3.827025824	0.000129701	295.4233537	45413564.87
chr14.101589306-104303490	105583.9858	3.826638639	0.000129905	282.2045881	39503177.95
chr14.100165746-101589306	105579.9204	3.826586999	0.000129932	282.1767257	39504036.19
chr14.99649754-99667687	105449.8075	3.825780482	0.000130358	281.6550746	39479714.37
chr14.99667687-100162377	105449.8075	3.825780482	0.000130358	281.6550746	39479714.37

chr14.100162377-100165746	105449.8075	3.825780482	0.000130358	281.6550746	39479714.37
chr14.48643111-48867571	115122.3177	3.825031689	0.000130755	293.6282938	45135800.31
chr14.48913499-49110731	115077.5769	3.824979918	0.000130783	293.5489207	45112919.08
chr12.80862862-81136206	253795.7099	3.824759114	0.0001309	431.5231533	149267222.1
chr14.49110731-49111449	115094.483	3.823745606	0.00013144	293.0046142	45210004.82
chr14.49111449-49616408	115094.483	3.823745606	0.00013144	293.0046142	45210004.82
chr14.49616408-52536512	115094.483	3.823745606	0.00013144	293.0046142	45210004.82
chr14.47132829-47136641	114624.7366	3.82182814	0.000132466	291.5463709	45066005.09
chr14.56565478-57007527	225.2274747	3.817382058	0.000134875	13.95441325	3635.223815
chr14.57007527-57238411	225.2274747	3.817382058	0.000134875	13.95441325	3635.223815
chr14.56355859-56565478	225.1951552	3.81714543	0.000135005	13.95103337	3635.061042
chr12.81799667-82129644	248342.5177	3.816600425	0.000135303	421.2063612	146422304.5
chr12.82129644-82233279	248342.5177	3.816600425	0.000135303	421.2063612	146422304.5
chr12.82233279-83480195	248342.5177	3.816600425	0.000135303	421.2063612	146422304.5
chr12.83480195-83676241	248342.5177	3.816600425	0.000135303	421.2063612	146422304.5
chr14.28235034-28295396	97497.58162	3.816256188	0.000135492	267.1356399	35584089.13
chr14.28295396-28614685	97497.58162	3.816256188	0.000135492	267.1356399	35584089.13
chr14.28614685-29067505	97497.58162	3.816256188	0.000135492	267.1356399	35584089.13
chr14.29067505-29731662	97497.58162	3.816256188	0.000135492	267.1356399	35584089.13
chr12.86438705-87177188	444207.2958	3.813400532	0.000137068	555.7892234	355026893.9
chr12.88453171-88723350	444203.6186	3.813382734	0.000137078	555.7696501	355033519.3
chr5.18642160-19586552	927894.7837	3.812774505	0.000137415	794.135594	1084183527
chr14.56267456-56355859	222.6967041	3.801337471	0.000143917	13.71627007	3615.693024
chr5.27672623-28007977	925972.5637	3.800569065	0.000144364	775.5449191	1105577727
chr5.17299011-17848822	844585.0614	3.77775654	0.000158247	710.886991	1003428020
chr5.17848822-17881818	844585.0614	3.77775654	0.000158247	710.886991	1003428020
chr5.17881818-18496350	844585.0614	3.77775654	0.000158247	710.886991	1003428020
chr5.6960825-7543807	886181.6703	3.770597182	0.000162857	717.7752704	1094100041
chr14.63394731-63698270	103995.2376	3.77030225	0.00016305	256.4222983	42176556.04
chr14.63150415-63394731	104239.5019	3.768954139	0.000163933	256.1603737	42418246.04
chr14.59312628-62756395	103765.1966	3.765983645	0.000165895	254.3922119	42325258.12
chr14.58455758-59312628	103459.0822	3.763877221	0.000167299	253.1791234	42277505.14
chr14.63698270-64446140	101559.3356	3.758657694	0.000170827	248.8570184	41446685.82
chr14.69985498-71117483	97229.30194	3.733611107	0.000188754	234.1243513	40378273.78
chr14.71117483-72281139	97229.30194	3.733611107	0.000188754	234.1243513	40378273.78
chr14.64446140-68010176	96583.21814	3.729572287	0.000191805	231.8661085	40231485.68
chr14.68010176-68358071	96583.21814	3.729572287	0.000191805	231.8661085	40231485.68
chr14.68358071-69922552	96583.21814	3.729572287	0.000191805	231.8661085	40231485.68
chr14.98456948-98937524	89947.14884	3.728470076	0.000192646	223.7687521	36155582.53
chr14.98937524-99044266	89947.14884	3.728470076	0.000192646	223.7687521	36155582.53
chr14.97359698-98456948	89641.01451	3.725546357	0.000194893	222.3586575	36137614.67

chr12.131934761-131938840	2147821.005	3.723487908	0.000196489	997.6321186	4624084353
chr12.131938840-132087415	2147821.005	3.723487908	0.000196489	997.6321186	4624084353
chr12.132087415-132377083	2147821.005	3.723487908	0.000196489	997.6321186	4624084353
chr14.74247410-74425056	91621.91095	3.710284021	0.000207027	219.1868849	38298708.3
chr14.72281139-73728957	91293.631	3.705601201	0.00021089	217.1539759	38380725.13
chr14.86817388-87022250	87123.78113	3.703049248	0.000213024	211.5448091	35881538.62
chr14.87022250-87113350	87123.78113	3.703049248	0.000213024	211.5448091	35881538.62
chr14.73728957-74247410	90092.17663	3.698967942	0.000216478	213.480379	38020357.32
chr14.87259377-87344148	86024.33731	3.697061972	0.000218109	208.2458911	35535810.91
chr14.87344148-90941592	86024.33731	3.697061972	0.000218109	208.2458911	35535810.91
chr1.194026432-194639330	2278584.785	3.694781892	0.000220076	966.3323611	5372839439
chr5.29541621-30630087	720640.6404	3.68596904	0.000227834	553.2944931	938601304
chr5.28007977-28496155	718403.5547	3.684160565	0.000229458	550.5483239	937435725.4
chr5.28496155-29541621	718403.5547	3.684160565	0.000229458	550.5483239	937435725.4
chr14.91163544-93140453	83619.5267	3.682368727	0.000231077	200.6219178	34852748.51
chr14.96139824-96556341	83377.77814	3.680892244	0.000232419	199.8663138	34782519.15
chr14.96556341-97203188	83377.77814	3.680892244	0.000232419	199.8663138	34782519.15
chr14.97203188-97359698	83377.77814	3.680892244	0.000232419	199.8663138	34782519.15
chr14.93140453-93908981	83043.22434	3.677958863	0.000235108	198.533654	34735557.27
chr14.93908981-94330704	83043.22434	3.677958863	0.000235108	198.533654	34735557.27
chr14.94330704-96112079	83043.22434	3.677958863	0.000235108	198.533654	34735557.27
chr1.194639330-195125690	2163845.196	3.677664735	0.000235379	909.8066463	5146396820
chr1.195815418-196983231	2515839.453	3.675308018	0.000237563	971.2570402	6516759099
chr1.193735519-194026432	2126306.779	3.672694551	0.000240006	892.967674	5063095397
chr14.90941592-90960503	81779.97685	3.672600365	0.000240095	195.3911275	34228599.32
chr14.90960503-91163544	81779.97685	3.672600365	0.000240095	195.3911275	34228599.32
chr1.195803021-195815418	2365504.579	3.656592956	0.00025559	906.6675378	6171624854
chr1.195387587-195418922	2022367.34	3.655706834	0.000256474	841.4876727	4860403536
chr1.195418922-195803021	2022367.34	3.655706834	0.000256474	841.4876727	4860403536
chr14.81673558-82561266	80376.35912	3.654077163	0.000258109	187.9874766	34365901.5
chr14.84050215-84305326	79846.08478	3.649519493	0.000262731	185.9997522	34276375
chr14.84305326-84323147	79846.08478	3.649519493	0.000262731	185.9997522	34276375
chr14.77138110-77496619	80785.22645	3.646733345	0.000265595	186.14435	35060171.38
chr1.193126047-193450741	2031703.237	3.646721248	0.000265608	827.2610244	4989740749
chr1.193450741-193735519	2031703.237	3.646721248	0.000265608	827.2610244	4989740749
chr14.83065713-83405239	79216.80693	3.645192514	0.000267192	183.9918358	34106418.2
chr14.83405239-83497296	79216.80693	3.645192514	0.000267192	183.9918358	34106418.2
chr14.83497296-84050215	79216.80693	3.645192514	0.000267192	183.9918358	34106418.2
chr14.76471997-77138110	80530.88978	3.644792086	0.000267608	185.2729724	35003617.23
chr14.82561266-83065713	79034.86826	3.643384938	0.000269076	183.2442464	34088439.46
chr14.99536104-99600519	75525.99587	3.639528104	0.000273138	178.294045	31993082.29

chr1.196983231-199628083	2268349.037	3.639038705	0.000273658	856.1767314	6009749112
chr1.199700152-200012511	2268340.078	3.639030788	0.000273666	856.1604903	6009815646
chr5.13843485-15003609	606345.5979	3.638412651	0.000274324	465.207361	790303453.8
chr14.99044266-99056323	75430.26675	3.638394119	0.000274343	177.8541447	31990961.77
chr14.99056323-99536104	75430.26675	3.638394119	0.000274343	177.8541447	31990961.77
chr1.200012511-200023377	2258218.363	3.636657736	0.000276199	850.0138014	5999373382
chr1.200023377-200240036	2258218.003	3.636657408	0.000276199	850.0131336	5999376183
chr14.74425056-76428857	78791.60974	3.634239892	0.000278801	180.211922	34448984.82
chr14.77496619-77582579	78456.97181	3.63240743	0.000280789	179.3082578	34329129.64
chr14.77582579-78004502	78456.97181	3.63240743	0.000280789	179.3082578	34329129.64
chr14.99600519-99649754	71855.90131	3.626677343	0.000287092	170.5635082	30271836.02
chr14.78004502-79818815	77013.49061	3.624657143	0.000289345	175.4887664	33797478.08
chr14.79818815-80100454	77013.49061	3.624657143	0.000289345	175.4887664	33797478.08
chr14.80100454-80209937	77013.49061	3.624657143	0.000289345	175.4887664	33797478.08
chr5.17072233-17299011	541550.0084	3.623998519	0.000290083	429.259366	683214939.2
chr14.86004208-86078161	75087.41811	3.62010364	0.000294485	172.139843	32753139.9
chr14.86078161-86817388	75087.41811	3.62010364	0.000294485	172.139843	32753139.9
chr14.84323147-85853080	75334.71763	3.613182741	0.000302461	170.4035261	33305177.49
chr12.132377083-132407202	1012073.249	3.585977836	0.000335817	528.4249465	1938387407
chr12.132407202-133161346	1012073.249	3.585977836	0.000335817	528.4249465	1938387407
chr14.85899888-86004208	71006.03426	3.585288831	0.000336705	158.2142545	31867273.38
chr5.30630087-30630563	577071.1753	3.581809887	0.000341222	406.1519113	819917701
chr5.914233-1952000	432067.1861	3.577587937	0.00034678	353.2988187	528397049.2
chr5.1952000-2295088	431684.8429	3.577009484	0.000347548	352.7516837	528280408.5
chr5.2295088-2473048	431684.8429	3.577009484	0.000347548	352.7516837	528280408.5
chr5.2473048-2782715	431684.8429	3.577009484	0.000347548	352.7516837	528280408.5
chr14.81374822-81673558	69288.70597	3.574901352	0.00035036	153.7221088	31231192.53
chr1.114669174-114698094	1601525.352	3.574332993	0.000351122	634.3296921	4043454824
chr1.113060233-114669174	1571804.207	3.567881413	0.000359879	620.1499247	3983824501
chr1.111585148-112357730	1571799.925	3.567876047	0.000359887	620.1418539	3983854644
chr1.112853144-113060233	1571796.816	3.567872059	0.000359892	620.1358679	3983877336
chr1.112357730-112853144	1571794.632	3.567869193	0.000359896	620.1315755	3983893840
chr14.80209937-80957294	67399.24395	3.557829906	0.000373931	147.4379831	30810636.38
chr14.80957294-81374822	67399.24395	3.557829906	0.000373931	147.4379831	30810636.38
chr1.111066532-111585148	1462911.331	3.54057347	0.000399258	565.3573906	3785410075
chr12.103238017-103420584	990663.1371	3.524290726	0.000424618	458.5760442	2140132403
chr12.84468344-84863708	120590.6553	3.522745943	0.000427101	179.5573704	80988633.93
chr1.115399940-115409612	1409249.818	3.519771243	0.000431919	530.8356365	3741242886
chr12.117078671-117423264	1580290	3.516144342	0.000437863	553.918777	4508452482
chr12.104638815-104848409	945738.8929	3.510038353	0.000448042	435.4829146	2053862559
chr5.7932409-8018960	408498.5162	3.507384118	0.000452535	298.9488347	558192635.1

chr5.8018960-8055995	408498.5162	3.507384118	0.000452535	298.9488347	558192635.1
chr12.103420584-104638815	938828.8464	3.506437831	0.000454148	430.6655747	2046598695
chr5.5675901-6272826	423188.6814	3.50512854	0.000456387	302.2337655	592550140.2
chr5.6272826-6607143	423188.6814	3.50512854	0.000456387	302.2337655	592550140.2
chr1.114698094-115399940	1338729.036	3.503740019	0.000458773	500.5760598	3580265968
chr5.98334408-99563090	0.164669406	-3.49928602	0.000466506	0.059956284	0.452263075
chr5.15003609-16834568	399942.6703	3.499063344	0.000466896	291.1366628	549412561.1
chr5.16834568-17072233	399942.6703	3.499063344	0.000466896	291.1366628	549412561.1
chr5.30630563-31589480	468828.3298	3.49794488	0.000468858	311.4860838	705649511.2
chr5.31589480-31671704	468828.3298	3.49794488	0.000468858	311.4860838	705649511.2
chr5.31671704-31841279	468828.3298	3.49794488	0.000468858	311.4860838	705649511.2
chr5.98199204-98334408	0.164966543	-3.493514957	0.000476706	0.060024989	0.453377183
chr5.92792468-93099461	0.164927548	-3.490560202	0.000482009	0.059951506	0.453718309
chr12.115551934-117078671	1458876.256	3.490399689	0.000482299	504.3562905	4219873870
chr5.93658221-94527675	0.164983024	-3.489050731	0.000484739	0.059956747	0.453983902
chr5.94527675-94875601	0.164983024	-3.489050731	0.000484739	0.059956747	0.453983902
chr5.39430978-39432927	374670.8127	3.488777723	0.000485234	276.9640738	506846306.7
chr5.39432927-39521490	374670.8127	3.488777723	0.000485234	276.9640738	506846306.7
chr5.39521490-50124856	374670.8127	3.488777723	0.000485234	276.9640738	506846306.7
chr12.117753737-118083390	3271450.622	3.487770993	0.000487065	714.1037795	14987162204
chr12.118083390-118087053	3244402.64	3.485152011	0.000491858	707.0209983	14888028100
chr12.54231537-54997372	464134.4319	3.485056994	0.000492032	301.8390778	713694106.4
chr12.54997372-55637505	464134.4319	3.485056994	0.000492032	301.8390778	713694106.4
chr5.7543807-7932409	398855.0019	3.475244823	0.000510387	276.7418079	574851027.2
chr12.99726603-101514633	218747.8705	3.471488945	0.000517581	211.3834755	226368833.9
chr12.101514633-101694894	218747.8705	3.471488945	0.000517581	211.3834755	226368833.9
chr12.95960628-97884138	218666.8683	3.471388124	0.000517775	211.3067838	226283314.1
chr12.97884138-99059457	218666.8683	3.471388124	0.000517775	211.3067838	226283314.1
chr12.99059457-99726603	218666.8683	3.471388124	0.000517775	211.3067838	226283314.1
chr12.55677756-55809968	442510.8655	3.470873782	0.000518768	286.9017649	682518862.1
chr5.6607143-6762770	397211.9108	3.470486242	0.000519517	273.5037853	576874290.6
chr5.31841279-33614836	433912.9878	3.470257361	0.00051996	284.091569	662745753.6
chr12.55809968-56999730	438637.7786	3.467842167	0.000524655	283.9788437	677526178.5
chr12.56999730-58925230	436959.9139	3.466945267	0.000526409	282.9681498	674754266.4
chr5.8055995-8637212	371924.1001	3.466850813	0.000526594	263.7729784	524418904
chr5.8637212-9883770	371924.1001	3.466850813	0.000526594	263.7729784	524418904
chr12.102290868-103238017	845986.9659	3.465542823	0.000529162	375.9224529	1903833998
chr5.11009585-12382370	391649.1239	3.46047817	0.000539217	266.1720162	576277846.3
chr5.12382370-13843485	391649.1239	3.46047817	0.000539217	266.1720162	576277846.3
chr5.2782715-3188096	332629.3906	3.457179397	0.000545861	246.2742553	449264627
chr5.3188096-3224616	332629.3906	3.457179397	0.000545861	246.2742553	449264627

chr12.13509249-14334825	0.065052868	-3.455875952	0.000548508	0.013810929	0.306414984
chr12.13493060-13509249	0.065153802	-3.45298729	0.000554415	0.013826598	0.30701824
chr12.104848409-105697567	780542.0233	3.446688385	0.000567503	348.0785145	1750311567
chr12.105697567-106682580	780542.0233	3.446688385	0.000567503	348.0785145	1750311567
chr12.13456826-13492116	0.066893968	-3.446006033	0.000568938	0.014365125	0.311504628
chr12.13492116-13493060	0.066893968	-3.446006033	0.000568938	0.014365125	0.311504628
chr12.14334825-14822855	0.065289099	-3.433411321	0.000596037	0.013749664	0.310019681
chr5.9883770-10279647	348355.1906	3.431164084	0.000600997	237.8400557	510222462.1
chr12.114775401-115408648	1186680.163	3.422935048	0.000619489	394.6258403	3568468320
chr5.6762770-6960825	358515.2152	3.422698349	0.000620028	236.477916	543531344.3
chr12.52925969-54231537	388800.9672	3.420797763	0.000624377	243.8168969	619998835.4
chr12.115408648-115495229	1164077.276	3.420286045	0.000625553	388.9786777	3483676565
chr12.115495229-115551934	1164077.276	3.420286045	0.000625553	388.9786777	3483676565
chr12.59222210-60173406	376897.626	3.416813853	0.000633586	238.5458248	595490701.5
chr12.113821525-114139719	1141240.406	3.411969279	0.000644954	378.2605014	3443208212
chr12.114139719-114579068	1138152.653	3.410700273	0.000647963	376.7002268	3438785987
chr12.114579068-114775401	1138152.653	3.410700273	0.000647963	376.7002268	3438785987
chr12.65258960-65376638	117826.1287	3.409321779	0.000651246	143.1748631	96965321.33
chr12.58925230-59222210	370867.7467	3.408977868	0.000652068	232.9389142	590467616.9
chr12.60173406-60176579	360362.6658	3.403034188	0.000666419	227.1734865	571639115.6
chr12.60176579-61707927	360362.6658	3.403034188	0.000666419	227.1734865	571639115.6
chr12.61707927-62824724	360362.6658	3.403034188	0.000666419	227.1734865	571639115.6
chr12.65424497-65612542	112703.6675	3.395506085	0.000685019	136.7236193	92903601.66
chr5.38064232-39048915	166982.3833	3.389593955	0.000699962	159.5024038	174813141.8
chr5.39048915-39267617	166982.3833	3.389593955	0.000699962	159.5024038	174813141.8
chr5.39267617-39391701	166982.3833	3.389593955	0.000699962	159.5024038	174813141.8
chr5.39391701-39430978	166982.3833	3.389593955	0.000699962	159.5024038	174813141.8
chr5.10667969-10677796	203800.8614	3.383131729	0.000716642	171.1596087	242667013.6
chr5.10677796-11009585	203800.8614	3.383131729	0.000716642	171.1596087	242667013.6
chr12.109060238-110201605	615930.705	3.380789822	0.000722778	271.1031272	1399359119
chr12.110201605-112168009	615930.705	3.380789822	0.000722778	271.1031272	1399359119
chr5.37962942-38064232	159286.864	3.373870121	0.000741193	151.3930381	167592284
chr12.41413067-42418116	123440.0164	3.373607836	0.0007419	135.9797518	112056666.1
chr12.39223566-40956101	72752.07349	3.372245997	0.000745578	108.67253	48704711.27
chr12.113155813-113465367	1023509.063	3.370949431	0.000749096	327.8361306	3195409855
chr12.113465367-113821525	1016134.538	3.370451241	0.000750452	326.4570504	3162833820
chr12.112356655-113155813	1021269.816	3.370015102	0.000751641	326.8059842	3191471658
chr12.87177188-88453171	121509.3905	3.369197805	0.000753873	133.887162	110275935
chr12.73997381-74147976	104481.5062	3.367685769	0.000758019	125.3146652	87111792.63
chr12.112168009-112356655	1176676.681	3.365883077	0.00076299	343.3167316	4032917375
chr5.37955073-37962942	153934.9577	3.360550039	0.000777874	145.1915661	163204873.7

chr12.50824204-52505526	402909.5553	3.360437246	0.000778192	216.7647538	748904547
chr12.52505526-52525780	402909.5553	3.360437246	0.000778192	216.7647538	748904547
chr12.52525780-52925969	402151.4424	3.35971754	0.000780222	216.2457454	747879604.7
chr12.106682580-109060238	583764.8569	3.356698	0.000788792	250.8142372	1358700415
chr12.44711555-45205405	110530.598	3.351396485	0.000804051	124.1516893	98403921.63
chr12.50320008-50824204	372950.5012	3.349229227	0.000810367	204.7053368	679474597.7
chr12.45630259-45764179	56347.01549	3.348781418	0.000811678	93.38916977	33997369.95
chr12.49022976-50320008	369832.5256	3.344761823	0.000823533	201.959702	677244498
chr12.70119398-70989877	101955.4088	3.342538924	0.000830157	117.9314358	88143634.55
chr5.33614836-34852521	143360.4791	3.333608674	0.000857272	133.2769567	154206904.6
chr5.34852521-35023786	143360.4791	3.333608674	0.000857272	133.2769567	154206904.6
chr5.35023786-35361047	143360.4791	3.333608674	0.000857272	133.2769567	154206904.6
chr5.35361047-35840121	143360.4791	3.333608674	0.000857272	133.2769567	154206904.6
chr5.35840121-36405462	143360.4791	3.333608674	0.000857272	133.2769567	154206904.6
chr5.36405462-36619731	143360.4791	3.333608674	0.000857272	133.2769567	154206904.6
chr5.36619731-37668532	143360.4791	3.333608674	0.000857272	133.2769567	154206904.6
chr5.37668532-37955073	143360.4791	3.333608674	0.000857272	133.2769567	154206904.6
chr12.48358716-49022976	351580.2842	3.330675832	0.000866354	191.6108565	645102780.3
chr5.10279647-10280346	177149.8135	3.330446208	0.000867069	144.4434606	217261870.6
chr5.10280346-10667969	177149.8135	3.330446208	0.000867069	144.4434606	217261870.6
chr12.70989877-73375045	95521.36995	3.320385153	0.000898933	109.7580141	83131352.07
chr12.73375045-73846359	95521.36995	3.320385153	0.000898933	109.7580141	83131352.07
chr12.73846359-73997381	95521.36995	3.320385153	0.000898933	109.7580141	83131352.07
chr5.3224616-3428965	227054.5781	3.314935566	0.000916643	154.6339265	333392435.9
chr5.3428965-4005736	227054.5781	3.314935566	0.000916643	154.6339265	333392435.9
chr5.4005736-4141931	227054.5781	3.314935566	0.000916643	154.6339265	333392435.9
chr12.81429964-81793463	55596.58636	3.30059427	0.000964803	84.59336929	36539275.36
chr12.81136206-81429964	55369.03689	3.297771393	0.000974554	83.98501658	36503299.89

Supplementary Table 5. Selection of Survival related DNA methylation sites

Gene	HR	Z	pValue	lower	upper
LRP12	4.32E+10	4.052586	5.06547E-05	3.10E+05	6.02E+15
NTSR1	4.87E+11	3.875795	0.000106277	5.98E+05	3.96E+17
BRDT	3.05E-03	-3.76707	0.000165175	1.50E-04	6.21E-02
INTS4L2	8.51E+03	3.765264	0.000166373	7.66E+01	9.45E+05
AQP11	3.50E+05	3.714899	0.000203285	4.16E+02	2.94E+08
B4GALT6	1.21E+42	3.682421	0.000231029	4.83E+19	3.02E+64
SNCA	2.80E+11	3.67824	0.000234849	2.23E+05	3.52E+17
MRGPRE	7.10E-04	-3.597037	0.000321863	1.37E-05	3.69E-02
CHST11	1.66E+45	3.535715	0.000406673	1.42E+20	1.94E+70
PTPRD	7.47E-02	-3.524579	0.000424156	1.77E-02	3.16E-01
ZNF660	2.05E+16	3.460425	0.000539324	1.18E+07	3.54E+25
ZFP28	3.61E+04	3.447217	0.000566392	9.25E+01	1.41E+07
DOK6	1.02E-01	-3.30986	0.000933428	2.63E-02	3.94E-01

Supplementary Table 6. Marker genes for the different clusters

	Biomarker		Biomarker		Biomarker
Cluster 1	HOXB3	Cluster 2	ARPP21	Cluster 3	MYCT1
	HOXB5		KCNE5		MARCO
	HOXA6		TMIGD2		EFCC1
	C3ORF80		PCBP3		PTK2
	HOXB2		ADAMTS15		PEAR1
	HOXA3		ZNF608		ASGR2
	IGSF10		DDIT4L		TIAM1
	TAS2R60		CD200		SLITRK5
	TAS2R41		KIF26A		HIST3H2A
	CT45A10		PDPN		RUFY4
	SAGE1		LHX6		SIGLEC9
	ADAMDEC1		UMODL1		SIGLEC1
	MAP7		C3ORF56		PLXNA1
	CT45A1		PURG		SGK1
	RNASE3		OR10H1		TUSC1
	AIFM3		ZSCAN23		CDC42BPA
	SULF2		CYP4F11		HBA1
	C1ORF127		ZNF107		HLA-DQB1
	DTNA		RUNX1T1		SORBS3
	SCHIP1		PRODH		PPFIA4
	SORCS2		ADGRL3		NMU
	TMEM52B		IKZF2		TREML1
	ZNF334		ACSM1		HLA-DRB1
	CLEC4A		MLNR		NHSL2
	TLR6		M1AP		SYTL4
	FGD4		ADAM22		KLRF2
	CYSLTR2		ZNF391		SIRPG
	NEK10		ST18		H1F0

TNFSF13	CD69	OASL
AL928654.4	ZNF439	KIR2DL4
PANK1	USP41	NUP62CL
CLEC2B	PLEKHA5	HELZ2
CD101	AGAP9	HLA-DPA1
MATN4	ADGRD2	GOLGA6L7P
TMEM65	OR9A4	PDZD4
ODF3B	LRP12	ZNF831
GPR141	TFF3	PI4K2A
MCTP1	BCL2L10	RTP5
PSRC1	ZNF154	IFIT1B
ITPRIPL2	IL9R	BICDL1
NHSL1	KCTD15	STARD8
RASSF8	CABLES1	MAN1C1
IFNK	AC092042.3	KIR3DL1
RPA4	CARNS1	NEURL1
FER1L6	NMRK2	MYL4
OR2B11	SIGLEC8	ANKRD65
SMCO2	SCART1	MMP17
HIST1H2BL	C16ORF96	NOXA1
FAM83A	C2ORF82	KLF12
PADI4	SSBP2	CD6
CCDC73	MST1	RTL8A
AL049634.2	PRSS12	HIST1H3E
SCG5	B4GALT6	PPM1N
CT45A5	DUSP15	KIR3DL2
ARNTL2	ZSCAN31	DYNC1I1
MARCH	KCNJ11	NAV1

CSPG4	ZNF660	HFE
OXER1	SYTL2	OVCH1
RNF208	METTL24	DTX1
B3GNT8	PGBD1	NPL
BTBD19	ZNF273	PLCG1
ASRGL1	ANKRD28	TCF15
PSTPIP2	IGFBP7	H2AFJ
CARD17	LPAR4	TNFSF8
TPK1	SHF	SOCS3
HIST1H2BC	TAS2R3	LGR6
SPDYE2B	RGS3	SELENOM
FUT4	GSDMB	TTC39B
HPD	FAM25G	TMPRSS4-AS1
ANKRD63	SLC29A4	CCR7
SLC4A7	TAS2R5	ETS1
RFLNB	GNG2	PDE1A
TNFSF13B	KLHL13	INPP4B
CLDN3	ZSCAN12	IFI27
GAPT	PRB3	IPCEF1
ADPRH	GOLGA8Q	MSC
DENND1A	NTRK3	GIMAP5
RNASE8	RASAL1	IFITM5
NUDT7	GALNT1	FAM69C
ASIP	OXTR	FBLN5
ZCWPW1	C12ORF71	CCL3L3
SNX13	MAP10	IFITM2
PLIN5	ERAS	ARSG
LRRC74A	SPINK8	ASTL
P4HA1	ZNF124	DPF3
CD300LD	CDADC1	CLEC1A

	FAM107B		CDH24		TNFRSF14
	ACOT4		SLC2A7		RHBDF1
	GDPD4		ZNF681		APOL3
	MINDY1		OR13D1		RAP1GAP
	PRDM2		RPUSD3		OXCT2
	HIST2H2BF		MXRA7		DPEP1
	TMCO4		GPR152		CCL25
	ATP8B3		ARHGEF28		NDRG2
	HIST1H2BN		MTCL1		FYN
	SH2B2		ZNF415		LY6G6E
	LMLN		AK9		TPD52
	RPA3		C9ORF152		RTL5
	SPACA5		IRGM		GP5
	AC040162.4		TEPP		UNC13B

Supplementary Table 7. AML Patients' information

Patient's Number	Gender	Age	Diagnosis	Blast cells Proportion	FAB Subtypes	Status
202431	Male	44	Acute Myeloid Leukemia	82.5	AML-M5	ND
202432	Female	65	Acute Myeloid Leukemia	80.5	AML-M1	ND
202433	Male	33	Acute Myeloid Leukemia	81.5	AML-M2	ND
202434	Male	33	Acute Myeloid Leukemia	91	AML-M1	ND
202435	Female	19	Acute Myeloid Leukemia	18.3	AML-M2	R/R
202509	Male	57	Acute Myeloid Leukemia	24	-	ND
202510	Female	53	Acute Myeloid Leukemia	87.19	AML-M4	ND
202511	Male	45	Acute Myeloid Leukemia	93	AML-M5	ND
202512	Male	76	Acute Myeloid Leukemia	54	AML-M2a	ND
202513	Male	51	Acute Myeloid Leukemia	68	AML-M2	ND

Supplementary Table 8. AUC of candidate drug in cluster 3 patients

**Candidate Drug**	**AUC (Mean ±SD)**
Elesclomol	42.09 ± 54.20
Bortezomib	150.9 ± 55.23
CI-1040	179.5 ± 45.36
Ruxolitinib	225.0 ± 35.66
Imatinib	225.2 ± 42.92
GSK-1904529A	235.6 ± 42.25
PHT-427	253.9 ± 47.06
Selumetinib	185.0 ± 57.24
